# Mating induces switch from hormone-dependent to hormone-independent steroid receptor–mediated growth in *Drosophila* secondary cells

**DOI:** 10.1371/journal.pbio.3000145

**Published:** 2019-10-07

**Authors:** Aaron Leiblich, Josephine E. E. U. Hellberg, Aashika Sekar, Carina Gandy, Claudia C. Mendes, Siamak Redhai, John Mason, Mark Wainwright, Pauline Marie, Deborah C. I. Goberdhan, Freddie C. Hamdy, Clive Wilson

**Affiliations:** 1 Department of Physiology, Anatomy and Genetics, University of Oxford, Oxford, United Kingdom; 2 Nuffield Department of Surgical Sciences, University of Oxford, Oxford, United Kingdom; Cornell University, UNITED STATES

## Abstract

Male reproductive glands like the mammalian prostate and the paired *Drosophila melanogaster* accessory glands secrete seminal fluid components that enhance fecundity. In humans, the prostate, stimulated by environmentally regulated endocrine and local androgens, grows throughout adult life. We previously showed that in fly accessory glands, secondary cells (SCs) and their nuclei also grow in adults, a process enhanced by mating and controlled by bone morphogenetic protein (BMP) signalling. Here, we demonstrate that BMP-mediated SC growth is dependent on the receptor for the developmental steroid ecdysone, whose concentration is reported to reflect sociosexual experience in adults. BMP signalling appears to regulate ecdysone receptor (EcR) levels via one or more mechanisms involving the EcR’s N terminus or the RNA sequence that encodes it. Nuclear growth in virgin males is dependent on ecdysone, some of which is synthesised in SCs. However, mating induces additional BMP-mediated nuclear growth via a cell type–specific form of hormone-independent EcR signalling, which drives genome endoreplication in a subset of adult SCs. Switching to hormone-independent endoreplication after mating allows growth and secretion to be hyperactivated independently of ecdysone levels in SCs, permitting more rapid replenishment of the accessory gland luminal contents. Our data suggest mechanistic parallels between this physiological, behaviour-induced signalling switch and altered pathological signalling associated with prostate cancer progression.

## Abstract 2

Hormone receptor-dependent growth of secondary cells in the accessory glands of male fruit flies switches to a hormone-independent mechanism after mating, reminiscent of changes seen in advanced prostate cancer in humans.

## Introduction

**Fig 1:**
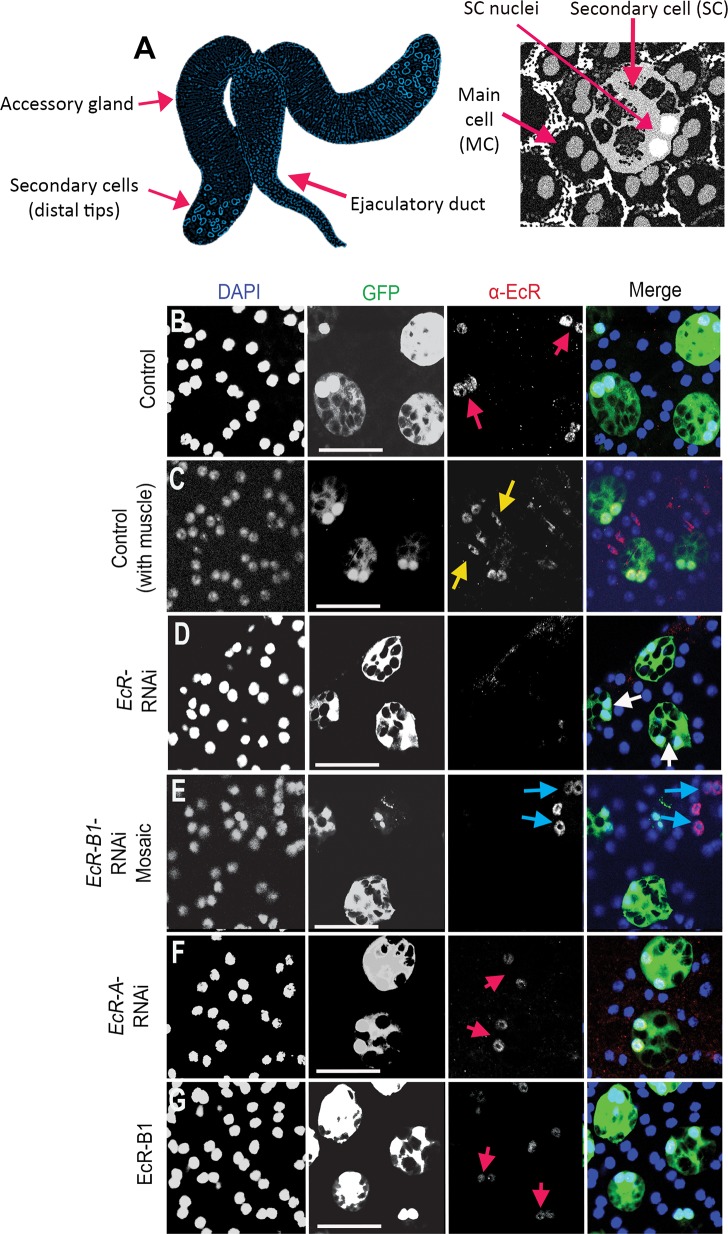
The EcR-B1 isoform of the EcR is the predominant isoform expressed in SC nuclei. (A) Schematic of *Drosophila* male AGs and binucleate SCs and MCs within their monolayer epithelium. (B-G) Images show distal tips of AGs dissected from 6-day-old males (except for mosaic in [E]). SCs (nuclei marked by red arrows) express nuclear GFP (which also labels SC cytosol) and other transgenes under esg^ts^F/O control. Nuclei are stained with DAPI (blue). (B, C) Immunostaining with an antibody that cross-reacts with all EcR isoforms reveals expression in SC nuclei ([B]; red arrows) and in muscle cell nuclei ([C]; yellow arrows) but not in MCs (non-GFP-positive nuclei in [B]) in 6-day-old males. (D-F) Whereas expression of an RNAi targeting the EcR-A transcript does not affect EcR expression (F), mosaic expression of an RNAi targeting *EcR-B1* transcripts ([E], green cells) or expression of an RNAi targeting all isoforms (D) strongly reduces nuclear EcR staining in SCs. In mosaics, staining is still present in non-RNAi-expressing SCs, which are not labelled with GFP ([E]; blue arrows). (G) esg^ts^F/O-driven expression of the EcR-B1 isoform has no detectable effect on nuclear EcR levels in SCs. Scale bars, 50 μm. AG, accessory gland; EcR, ecdysone receptor; *esg*, *escargot*; esg^ts^F/O, the yeast transcription factor GAL4 expressed under the control of the promoter of the gene *esg* in a temperature-dependent fashion; GFP, green fluorescent protein; MC, main cell; RNAi, RNA interference; SC, secondary cell.

In all higher organisms in which fertilisation takes place in the female reproductive tract, males not only deliver sperm to females but also transfer seminal fluid containing a cocktail of molecules that optimise fecundity. For example, secretions from the mammalian prostate and seminal vesicles contribute most of the seminal fluid volume, activate sperm [1], and promote embryo implantation [2]. The paired accessory glands (AGs) in the fruit fly, *Drosophila melanogaster*, perform related functions and can also substantially alter female behaviour after mating, increasing egg laying, promoting sperm storage, and reducing female receptivity to subsequent mating attempts [3–6]. Most of the key AG proteins (Acps) involved, such as Sex Peptide, which plays a central role in driving female postmating responses, are secreted by about 1,000 so-called main cells (MCs) found in the monolayered AG epithelium [7,8]. However, secondary cells (SCs), a small population of about 40 epithelial cells at the distal tip of each AG (Fig 1A), also play an essential role [9–11].

As humans age, the prostate epithelium frequently becomes hyperplastic. Indeed, many males over the age of 65 develop symptomatic benign prostatic hyperplasia [12]. SCs are not proliferative, but they also grow in adults, unlike other cells in the AG [9]. Mating enhances this growth, which is most easily assayed by measuring nuclear size. Interestingly, we have found that autocrine bone morphogenetic protein (BMP) signalling is crucial for the normal, age-dependent growth of SCs both in virgin and mated males [9]. Growth of SCs involves elevated synthesis of macromolecules including secreted proteins. Inhibition of BMP signalling specifically in adult SCs reduces the ability of males to suppress female remating. Furthermore, BMP signalling promotes secretion of the contents of large compartments that contain so-called dense-core granules at their centre [13] and exosomes, nanovesicles formed inside endosomal compartments that are released by fusion of these compartments to the plasma membrane [14]. These exosomes appear to be involved in female behavioural reprogramming, providing at least part of the explanation for SCs’ BMP-dependent effects on females.

Although BMP signalling is implicated in mammalian prostate development [15] and cancer growth and metastasis [16,17], steroid signalling through the androgen receptor (AR) is thought to be the central regulator of these processes and prostate hyperplasia [18,19]. Aberrations in steroid signalling are implicated in both benign and malignant disease of this organ [20,21]. Because androgen levels are modulated by factors such as developmental stage [18], nutrition [22], and sexual activity [23], this endocrine input potentially allows males to adapt prostate function during development and in response to the environment and reproductive demands. In advanced cancer, hormone deprivation therapy effectively blocks tumour growth, but typically within 2 years, hormone-independent cells emerge, which frequently still require the AR for growth and will lead to the death of the patient [24].

Flies employ a more limited range of steroid hormones than mammals, with the major characterised steroid hormone, 20-hydroxyecdysone (usually called 20-HE or ecdysone), primarily involved in developmental transitions, particularly during metamorphosis [25,26]. However, ecdysone levels also fluctuate in adult males in response to sociosexual interactions [27]. Ecdysone regulates male courtship behaviour [28–30] and affects the male germ line [31,32]. Ecdysteroids can also induce expression of multiple Acps in AGs [33], and the ecdysone receptor (EcR) is required for normal AG development [34]. However, the cells and molecular mechanisms involved in these processes, as well as the physiological functions of the EcR in adult AGs, remain unclear. We hypothesised that ecdysone signalling might affect adult SC function, potentially providing a sociosexual environmental input, which complements mating-dependent growth of SCs.

Here, we show that the EcR is specifically expressed in SCs within the adult AG epithelium and that EcR signalling in these cells is critical for normal growth. BMP signalling promotes growth by regulating levels of the EcR protein. Whereas nuclear growth in virgin males is BMP, EcR, and ecdysone dependent, some EcR-mediated nuclear growth observed after mating is hormone independent and specifically drives endoreplication in a subset of SCs, increasing their secretory activity. We propose that this novel form of steroid receptor control in flies permits SC activity to be flexibly regulated following mating to ensure that resources are employed according to demand.

## Results

### EcR-B1 is the predominant isoform expressed by SCs in the adult AG epithelium

In order to mark and genetically manipulate SCs, we used the SC-specific esg^ts^F/O GAL4 system [35], in which the yeast transcription factor GAL4 can be expressed in SCs under the control of the promoter of the gene *escargot* (*esg*) in a temperature-dependent fashion. Expression can be activated exclusively in adults through inactivation of a ubiquitously expressed, temperature-sensitive form of the GAL4 inhibitor GAL80 (tub-GAL80^ts^) by a temperature shift to 28.5°C at eclosion [9,35]. Staining AGs with a pan-EcR antibody that cross-reacts with all three characterised EcR isoforms [36] revealed EcR expression in the nuclei of muscle cells and SCs, which (like MCs) are binucleate (Fig 1B and 1C). This staining was lost in SCs expressing a previously characterised RNA interference (RNAi) construct targeting transcripts for all EcR isoforms (Fig 1D) [37].

We were unable to detect a robust signal in the AG with isoform-specific EcR-A and EcR-B1 antibodies. However, in an alternative approach, we expressed isoform-specific RNAi constructs [38] in SCs under esg^ts^F/O control, either throughout adulthood or by using a temperature shift in 3-day-old males. The latter approach leads to maintained RNAi expression in only a subset of SCs [9]. *EcR-A*-RNAi did not affect EcR levels (Fig 1F), but little, if any, EcR protein was detected in SCs expressing an *EcR-B1*-RNAi construct, demonstrating that EcR-B1 is the predominant isoform produced by these cells (Fig 1E).

### At least two EcR isoforms promote hormone-dependent SC nuclear growth in virgin males

To test the roles of the EcR in adult SC growth, the pan-*EcR*-RNAi construct was expressed in these cells posteclosion. As described previously, we assessed growth by measuring SC nuclear size in adult virgin males after 6 days relative to nuclear size in adjacent MCs, which do not grow with age or in response to mating, because this controls for nuclear size changes produced by flattening AGs upon mounting [9].

**Fig 2:**
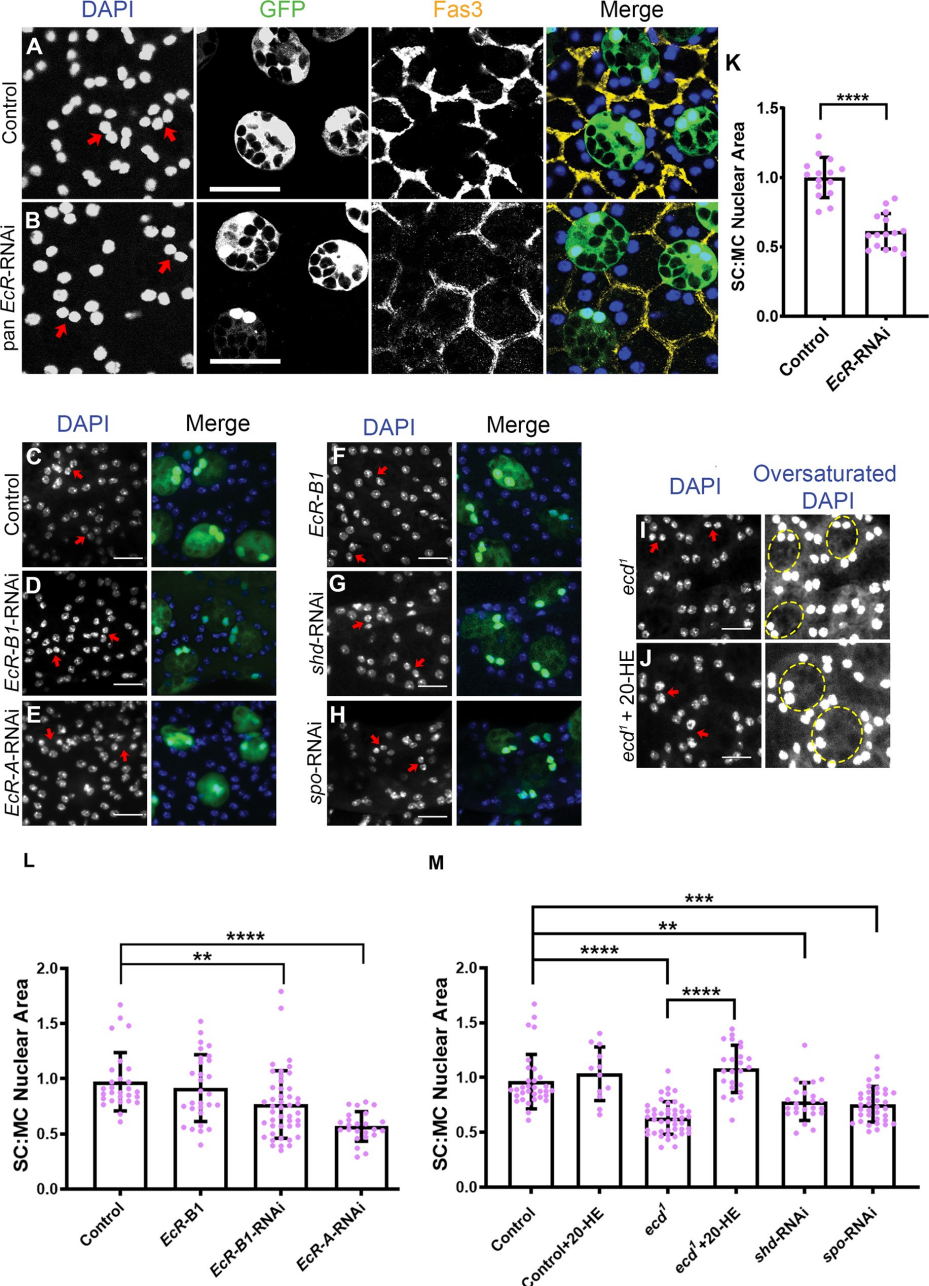
Ecdysone and the EcR are required to promote SC nuclear growth in virgin males. (A-J) Dissected AGs from 6-day-old virgin males were stained with DAPI (blue nuclei) and, in some cases (A and B), with an antibody against Fas3 to label the apical outlines of SCs and neighbouring MCs (yellow). Selected SC nuclei are marked with red arrows, and in (A-H), express GFP and other transgenes under esg^ts^F/O control. In *ecd*^*1*^ flies, SCs are recognised by their characteristic vacuolar morphology; selected SCs are highlighted by dashed circles (I and J). (A-E) RNAi knockdown of *EcR* expression in SCs with a pan-*EcR*-RNAi (B), *EcR-B1*-RNAi (D), or *EcR-A*-RNAi (E) significantly restricts growth of SC nuclei compared with control glands expressing GFP only (A and C). (F) Overexpression of EcR-B1 in SCs has no effect on SC nuclear size. (G, H) SCs expressing RNAis targeting the ecdysone synthesis genes *shd* (G) and *spo* (H) have smaller nuclei. (I, J) The SC nuclei of the temperature-sensitive *ecd*^*1*^ mutant are significantly smaller than control glands when adult virgin males are maintained at 28.5°C (I), which blocks *ecd* function, but not when these flies are fed with 20-HE (J). (K-M) Histograms showing size of SC nuclei relative to MC nuclei in AGs in which SCs express different transgenes and in *ecd*^*1*^ mutant males. Significance was assessed by an unpaired *t* test (K), Kruskal-Wallis test with Dunn’s multiple-comparison test (L), or one-way ANOVA with Sidak’s multiple-comparison test (M). *p < 0.05, **p < 0.01, ***p < 0.001, ****p < 0.0001, *n* = 15 (K), *n* ≥ 24 (L, M). Scale bars, 50 μm (A, B), 20 μm (C-J). Underlying data for this figure can be found in S1 Data. 20-HE, 20-hydroxyecdysone; AG, accessory gland; *ecd*, *ecdysoneless*; EcR, ecdysone receptor; *esg*, *escargot*; esg^ts^F/O, the yeast transcription factor GAL4 expressed under the control of the promoter of the gene *esg* in a temperature-dependent fashion; Fas3, Fasciclin3; GFP, green fluorescent protein; MC, main cell; RNAi, RNA interference; SC, secondary cell; *shd*, *shade*; *spo*, *spook*.

The growth of SC nuclei was inhibited by expression of *EcR*-RNAi (Fig 2A, 2B and 2K, S1 Data) but not by expression of a control RNAi targeting the *rosy* (*ry*) gene (S1H and S1I Fig, S1 Data). Surprisingly, not only did the isoform-specific *EcR-B1*-RNAi inhibit nuclear SC growth, but so did *EcR-A*-RNAi (Fig 2C–2E and 2L, S1 Data), suggesting that *EcR-A* is involved in this process despite its low levels of protein expression in SCs relative to EcR-B1. Because the EcR-B2 isoform only has a short unique N-terminal domain (NTD), there is no EcR-B2-specific RNAi available. EcR-B2’s function was tested by overexpressing a dominant negative form of the receptor that cannot bind ligand (EcR-B2-ΔC655.W650A) [49]; it did not affect SC nuclear growth (S1J Fig, S1 Data).

Steroid receptors typically modulate gene expression in the presence of their ligands, but in some contexts, it has been proposed that the unliganded EcR is repressive, and this repression is released by the hormone [39]. To test whether ecdysone is required to induce EcR-dependent growth in SCs or inhibit it, we initially employed a temperature-sensitive allele of *ecdysoneless* (*ecd*), *ecd*^*1*^ [25], a gene with pleiotropic effects on processes required for ecdysone synthesis and signalling in flies [40]. When males were shifted to the nonpermissive temperature (28.5°C) directly after eclosion, SCs and their nuclei failed to grow (Fig 2I and 2M, S1 Data), mirroring the phenotype of *EcR* knockdown. This growth suppression was rescued by feeding exogenous 20-HE to adult virgin *ecd*^*1*^ males (Fig 2J and 2M, S1 Data), confirming that reduced ecdysone levels in *ecd*^*1*^ flies are responsible for the phenotype.

Insect adult AGs can produce ecdysone, and SCs have previously been proposed to be sites of ecdysone synthesis in *Drosophila* [41] because they express Torso, the receptor for prothoracicotropic hormone (PTTH), which stimulates ecdysone biosynthesis in the larval ring gland. We knocked down in SCs two genes in the ecdysone synthesis pathway: *shade* (*shd*), which is involved the final step in the pathway, and *spook* (*spo*), which encodes a cytochrome P450 enzyme that appears to function in nonlarval stages [42]. Adult SC growth was inhibited after both treatments (Fig 2G, 2H and 2M, S1 Data). These experiments strongly support the hypothesis that ecdysone synthesised in SCs is required for normal EcR-dependent SC growth in virgin males.

In several other cell types, the EcR functions as a heterodimer with the nuclear receptor Ultraspiracle (USP) [43,44]. USP can promote nuclear localisation of EcR in Chinese hamster ovary (CHO) cells [45]. Staining with an antibody that recognises USP [46] revealed that this protein is expressed in SC nuclei (S2H Fig). However, *Usp*-RNAi knockdown in SCs had no effect on cell growth (S1A, S1B and S1I Fig, S1 Data) or EcR localisation and expression (S2A and S2B Fig) in SCs, even though it strongly reduced USP levels (S2I Fig), suggesting that conventional EcR/USP-mediated transcriptional regulation does not drive SC growth in virgin males.

### The EcR promotes hormone-independent SC nuclear growth in mated males

**Fig 3:**
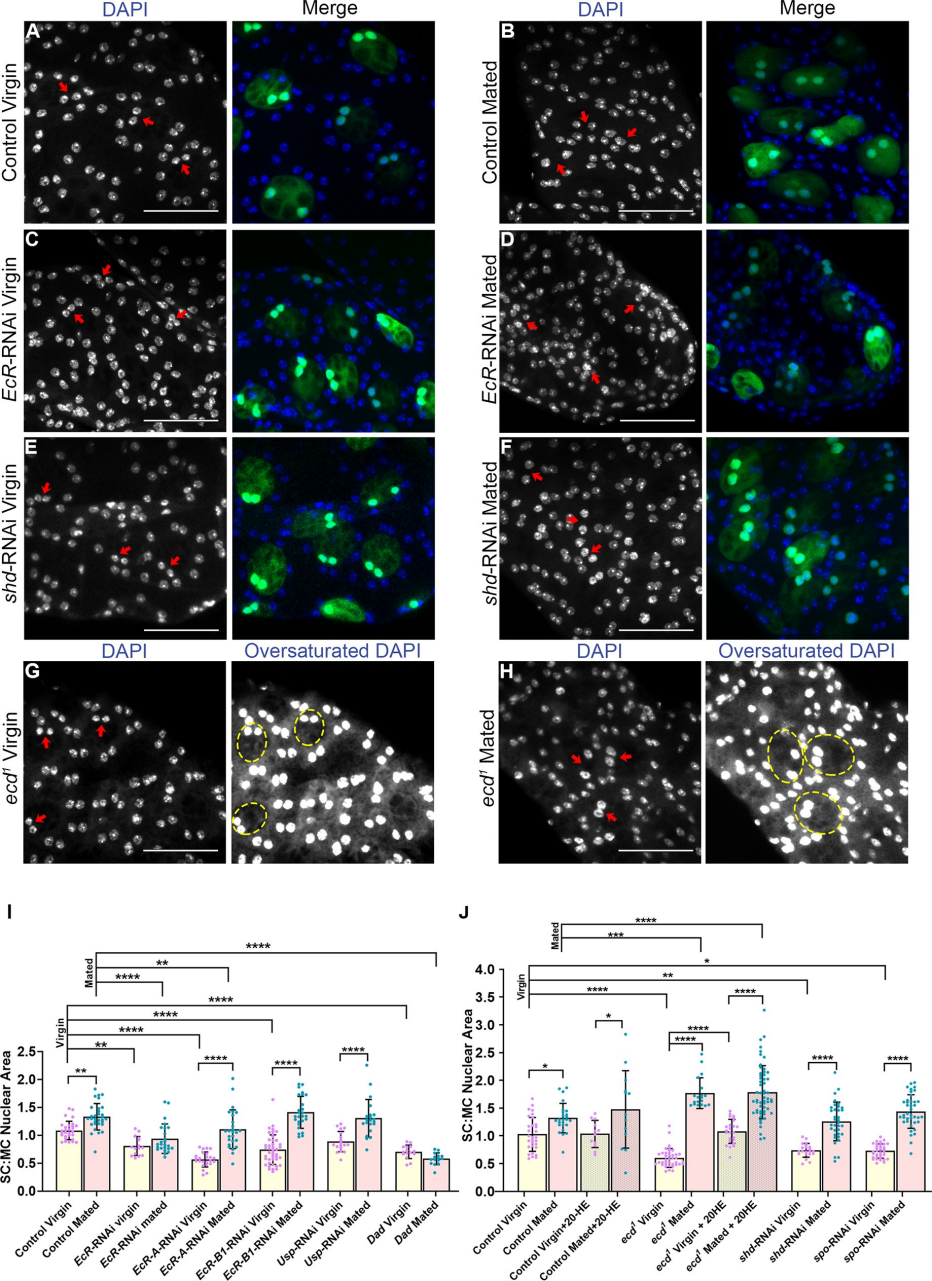
Mating induces hormone-independent, EcR-regulated SC nuclear growth. (A-H) SCs from glands of 6-day-old males were identified by GFP expression or their characteristic vacuolar morphology in *ecd*^*1*^ flies. In the latter, the approximate outlines of selected SCs are marked by a dashed circle. (A-D) SC nuclei (marked by red arrows; stained with DAPI) following pan-*EcR* knockdown are smaller than controls in virgin ([C] versus [A]) and mated ([D] versus [B]) males. (E, F) Knockdown of *shd* in SCs only suppresses nuclear growth in virgin males. (G, H) In *ecd*^*1*^ virgin males (G), nuclei are much smaller than controls, but they are enlarged compared with controls after mating (H). (I, J) Histograms showing SC nuclear size relative to adjacent MC nuclei in 6-day-old males for control glands, glands expressing different transgenes in SCs under esg^ts^F/O control, and *ecd*^*1*^ mutant glands (with and without 20-HE in the diet). Mating induces nuclear growth in control glands (B), which is suppressed when EcR and BMP signalling is reduced (I). Nuclear size is unaffected by 20-HE feeding in mated control and *ecd*^*1*^ mutant males (J). Data in (I) and (J) were analysed using one-way ANOVA and Sidak’s multiple-comparisons test; *p < 0.05, **p < 0.01, ***p < 0.001, ****p < 0.0001. *n* ≥ 15. Scale bars, 50 μm. Underlying data for this figure can be found in S1 Data. 20-HE, 20-hydroxyecdysone; BMP, bone morphogenetic protein; Dad, Daughters against Decapentaplegic; *ecd*, *ecdysoneless*; EcR, ecdysone receptor; *esg*, *escargot*; esg^ts^F/O, the yeast transcription factor GAL4 expressed under the control of the promoter of the gene *esg* in a temperature-dependent fashion; GFP, green fluorescent protein; MC, main cell; RNAi, RNA interference; SC, secondary cell; *shd*, *shade*; *spo*, *spook*; USP, Ultraspiracle.

To test whether EcR and ecdysone signalling are also required for the additional growth of SC nuclei observed in multiply-mated males [9], we cultured individual newly eclosed males with 7–10 virgin females for 6 days and then analysed nuclear size. Pan-*EcR* knockdown strongly suppressed nuclear growth under these conditions, mirroring the effects of blocking BMP signalling by expressing the transcriptional repressor Daughters against Decapentaplegic (Dad) (Fig 3A–3D and 3I, S1 Data) [13]. Whereas expression of *EcR-B1*-RNAi in mated males did not inhibit SC nuclear growth, *EcR-A*-RNAi did (Fig 3I, S1 Data), suggesting that it plays a more important role than *EcR-B1* after mating. Knockdown of *Usp* also had no effect on SC nuclear growth (Fig 3I, S1 Data). These experiments again suggest that a USP-independent form of EcR signalling controls SC nuclear growth in mated males, but unlike in virgins, EcR-B1 is not required.

Surprisingly, when we analysed temperature-shifted *ecd*^*1*^ males, they exhibited higher levels of SC growth after mating than mated controls (Fig 3G, 3H and 3J, S1 Data). Feeding with 20-HE did not affect this overgrowth phenotype (Fig 3J, S1 Data). To further confirm that EcR-associated SC nuclear growth in mated males is ecdysone-independent, the effects of *shd* and *spo* knockdown in SCs were also examined. Again SC nuclear growth was not suppressed by these treatments (Fig 3E, 3F and 3J, S1 Data). This suggests that, unlike in virgin males, EcR-mediated nuclear growth in response to mating is primarily hormone independent.

One possible explanation for this switch to hormone-independent growth is that it allows SCs to rapidly increase their biosynthetic capacity after mating independently of ecdysone levels. Ecdysone is thought to fluctuate in adult males according to sociosexual experience, so SC growth in virgins could vary in response to this. For example, work by Ishimoto and colleagues [27] has previously shown that ecdysteroid synthesis is increased in males immediately after they have been rejected by females over a 7-hour period.

We therefore tested whether SC growth is enhanced in rejected virgin males. However, because males must age for 6 days in order to observe sizeable increases in SC nuclear growth, we exposed males to repeated daily rejection over a 6-day period (see Materials and methods). We did not detect any difference in SC growth in adult males subjected to this ‘rejection regime’ when compared with males cultured in the absence of females (S3A Fig, S1 Data). However, we were also unable to detect any change in 20-HE concentrations in the former males (S3B Fig, S1 Data), providing an explanation for this finding. By contrast, 20-HE levels were significantly elevated in multiply-mated males compared with virgins (S3B Fig, S1 Data), suggesting that our assay could detect physiological changes in 20-HE concentration. We conclude from our mutant and knockdown analysis that in virgin males, but not in mated males, ecdysone levels can affect SC growth, but using the rejection assay, we were unable to detect long-term changes in 20-HE of sufficient magnitude to test the physiological implications of this regulation in virgins.

### EcR protein levels are controlled by BMP signalling to regulate growth

Because EcR-B1 is the major isoform expressed by SCs, we examined the effect of overexpressing it in these cells under esg^ts^F/O control. Unexpectedly, SC nuclear size was not affected by this treatment (Fig 2F and 2L, S1 Data) or by overexpression of EcR-A or EcR-B2 (S1D, S1F and S1I Fig, S1 Data). However, when we analysed EcR protein levels in these backgrounds, they appeared unchanged compared with controls (Fig 1G, S2A, S2D and S2F Fig), even though these constructs increase EcR levels when expressed in adjacent MCs (S4A, S4C, S4E and S4G Fig). Because many other UAS-coupled transgenes can be overexpressed in SCs under esg^ts^F/O control [9,13,14], our data suggest that EcR levels are tightly controlled by a mechanism that acts after the initiation of transcription in these cells so that increased *EcR* transcription has no obvious effect on receptor levels or growth.

**Fig 4:**
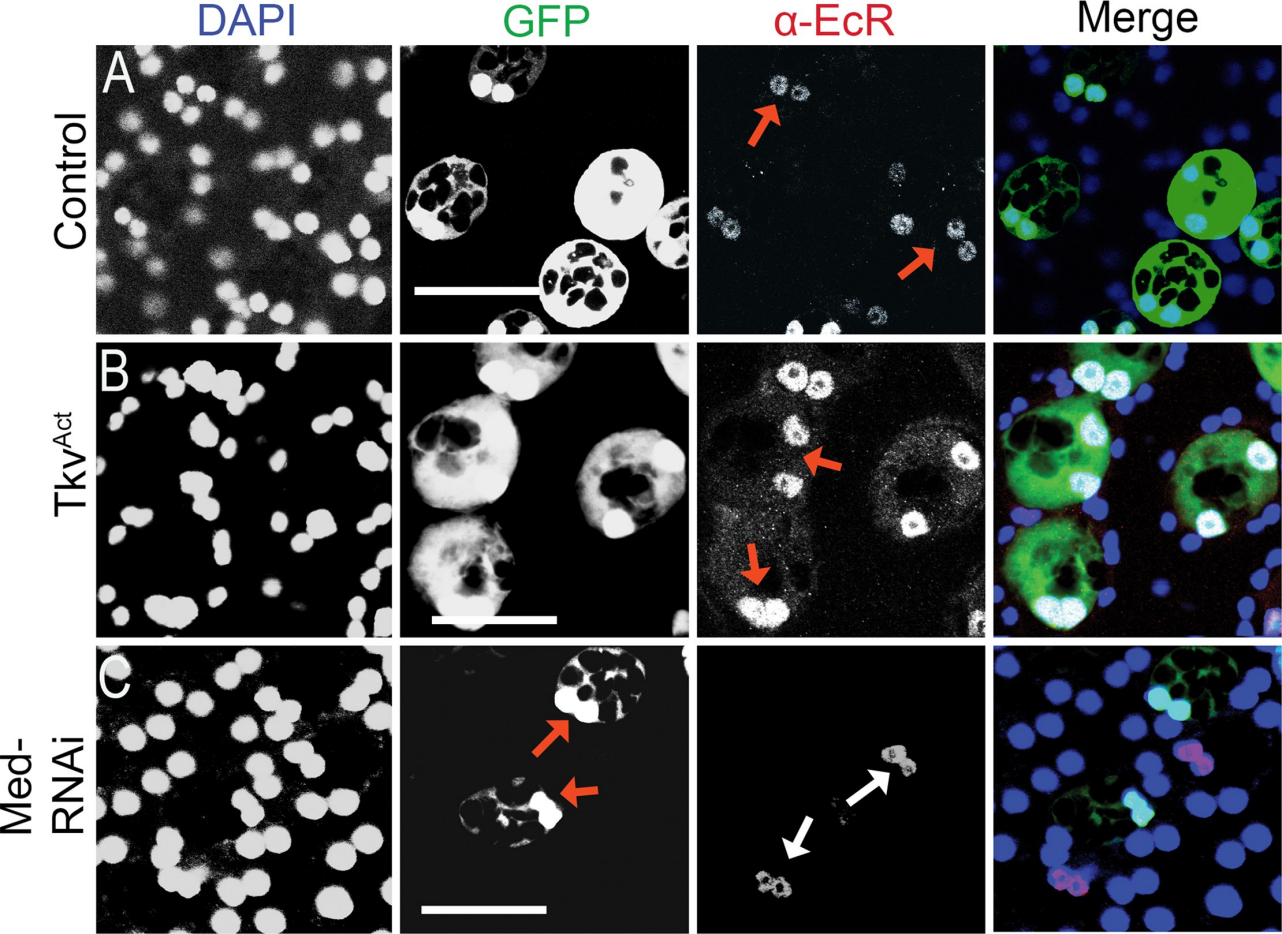
BMP signalling regulates levels of the EcR protein in SCs. Images show the AG epithelium dissected from 6-day-old virgin males expressing GFP and other transgenes under esg^ts^F/O control and stained with a pan-EcR antibody. (A, B) Up-regulation of BMP signalling by SC-specific Tkv^ACT^ overexpression in adults results in increased expression of EcR (B) compared with control (A), with an enhanced nuclear signal (red arrows) and some cytosolic expression. (C) RNAi knockdown of Med in only some SCs by activating the esg^ts^F/O driver system in 3-day-old adults reduces BMP signalling in these cells and leads to a marked reduction in SC-specific EcR expression (nuclei marked with red arrows) after a further 6 days. EcR expression in SCs that do not express the RNAi construct is normal (white arrows). Scale bars, 50 μm. AG, accessory gland; BMP, bone morphogenetic protein; EcR, ecdysone receptor; *esg*, *escargot*; esg^ts^F/O, the yeast transcription factor GAL4 expressed under the control of the promoter of the gene *esg* in a temperature-dependent fashion; GFP, green fluorescent protein; Med, Medea; RNAi, RNA interference; SC, secondary cell; Tkv, Thick veins.

Previous work has shown that SC growth is positively regulated by BMP signalling [9]. Because EcR signalling also promotes growth, we investigated the effect of BMP signalling on EcR protein expression. SC-specific expression of a constitutively active form of the type I BMP receptor Thick veins (Tkv^Q199D^ or Tkv^ACT^) [47] induced increased levels of EcR protein, which was primarily localised in the nucleus (Fig 4A and 4B). When BMP signalling was reduced in SC mosaics by inducing SC-specific knockdown of *Medea* (*Med*), encoding a downstream co-Smad transcription factor in the BMP signalling pathway, virtually no EcR protein was observed in knockdown cells (Fig 4C). A similar BMP-dependent effect on EcR levels was not observed in MCs, and MC growth was also unaffected by Tkv^ACT^, EcR, or combined Tkv^ACT^/EcR expression (S4 Fig). Taken together, these data indicate that BMP signalling is a key regulator of EcR protein levels specifically in SCs.

**Fig 5:**
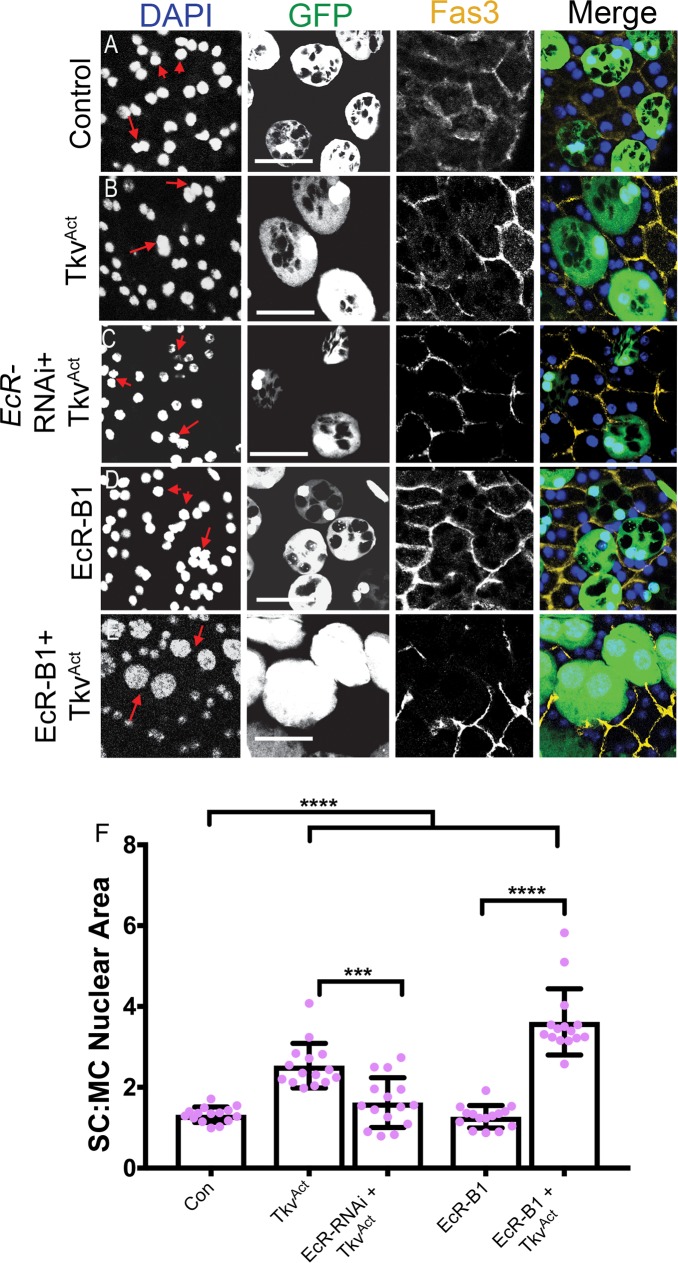
BMP signalling and the EcR synergise to regulate SC growth. Dissected AGs from 6-day-old virgin males expressing GFP and other transgenes under esg^ts^F/O control were stained with an antibody against Fas3 to mark the apical outlines of SCs and neighbouring MCs (yellow) and DAPI (blue nuclei). (A-C) The nuclear growth induced by SC-specific expression of Tkv^ACT^ (B) is completely suppressed by coexpression of pan-*EcR*-RNAi (C) to levels comparable with controls (A). (D, E) Coexpression of Tkv^ACT^ (to up-regulate BMP signalling) and EcR-B1 (E) produces a synergistic enhancement of nuclear (and cell) growth in 6-day-old adults relative to the effects of Tkv^ACT^ (B) or EcR-B1 (D) alone. Note that some MCs are compressed between the giant coexpressing SCs. (F) Histogram showing size of SC nuclei relative to MC nuclei in AGs in which SCs are expressing *Tkv* and *EcR* transgenes. Selected SC nuclei are marked with red arrows. Data were analysed using one-way ANOVA and Tukey’s multiple-comparisons test. *p < 0.02, ***p < 0.0001. *n* = 15. Scale bars, 50 μm. Underlying data for this figure can be found in S1 Data. AG, accessory gland; BMP, bone morphogenetic protein; Con, control; EcR, ecdysone receptor; *esg*, *escargot*; esg^ts^F/O, the yeast transcription factor GAL4 expressed under the control of the promoter of the gene *esg* in a temperature-dependent fashion; Fas3, Fasciclin3; GFP, green fluorescent protein; MC, main cell; RNAi, RNA interference; SC, secondary cell; Tkv, Thick veins.

To interrogate the interaction between BMP and EcR signalling further, we next tested whether overexpressing EcR when BMP signalling is hyperactivated might further increase EcR protein levels and promote growth. Co-overexpression of Tkv^ACT^ and EcR-B1 in SCs resulted in a strong synergistic enhancement of growth (Fig 5A, 5B and 5D–5F, S1 Data). Similar synergistic growth effects were observed with both EcR-A and EcR-B2, even though overexpressing either EcR isoform in the absence of Tkv^ACT^ had no effect on nuclear size (S1A, S1C–S1G and S1I Fig, S1 Data).

**Fig 6:**
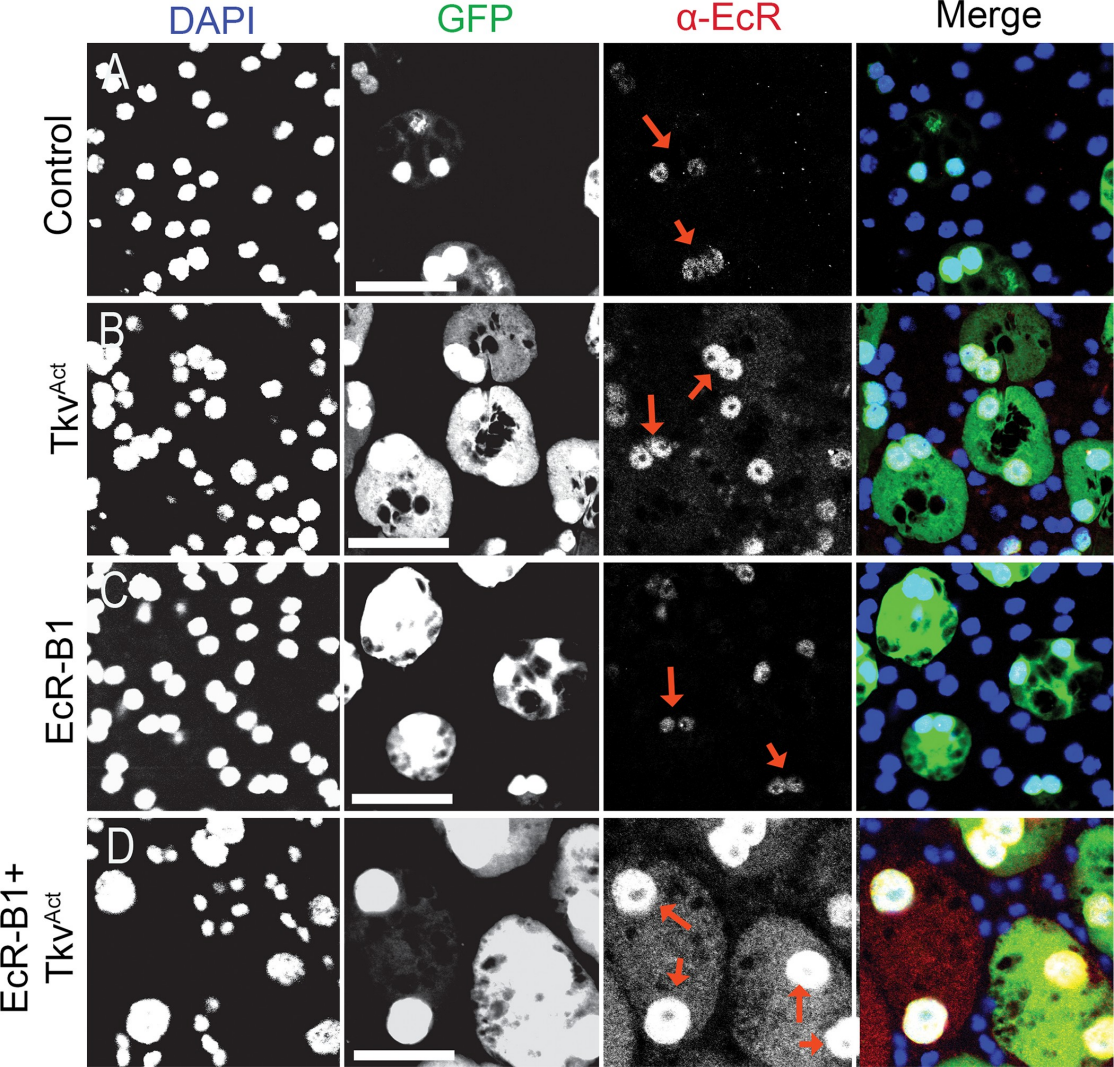
BMP signalling regulates EcR levels in SCs. Dissected AGs from 6-day-old virgin males expressing GFP and other transgenes under esg^ts^F/O control were stained with a pan-EcR antibody (red arrows) and DAPI (blue nuclei). (A, B) Nuclear EcR levels in SCs are elevated when BMP signalling is increased upon expression of Tkv^ACT^ (B) compared with controls (A). (C, D) Nuclear EcR levels are unaffected by overexpression of EcR-B1 alone (C), but levels of nuclear protein are highly up-regulated when coexpressed with Tkv^ACT^ (D). Some cytosolic EcR is also present. Scale bars, 50 μm. AG, accessory gland; BMP, bone morphogenetic protein; EcR, ecdysone receptor; *esg*, *escargot*; esg^ts^F/O, the yeast transcription factor GAL4 expressed under the control of the promoter of the gene *esg* in a temperature-dependent fashion; GFP, green fluorescent protein; SC, secondary cell; Tkv, Thick veins.

In addition to these dramatic growth effects, very high levels of EcR expression were observed in SCs coexpressing Tkv^ACT^ and any of the EcR isoforms (Fig 6, S2A and S2D–S2G Fig). The highest EcR levels were observed in nuclei, but all coexpressing cells also had detectable cytosolic EcR, which was highly elevated in some cells (Fig 6D, S2E and S2G Fig).

The increased expression in response to elevated BMP signalling could either result from greater transcript levels, posttranslational regulation, or both. When EcR isoforms were overexpressed in SCs, it was not possible to detect any increase in *EcR* transcripts using quantitative reverse-transcription PCR (RT-PCR) of AG mRNA. However, because muscle cells in the gland also express the *EcR*, this expression might mask the GAL4-induced increase in *EcR* transcripts in the small number of SCs in each gland, so we cannot conclude that transcript levels are unaffected by overexpression.

In light of the strong dependence of growth-regulatory EcR protein levels on BMP signalling, we tested whether BMP-dependent growth in SCs is mediated through EcR signalling by coexpressing Tkv^ACT^ with pan-*EcR*-RNAi. Tkv^ACT^-induced growth was strongly suppressed, and the resulting SC nuclei were not significantly different in size from wild-type controls (Fig 5A–5C and 5F, S1 Data), indicating that BMP-dependent SC growth requires the presence of the EcR.

### Sequences associated with the unique activation function 1–containing NTDs of each EcR isoform appear to be involved in SC-specific regulation of EcR levels by BMP signalling

Previous in vitro studies have revealed that when the *Drosophila* EcR-A and EcR-B1 isoforms are expressed in CHO cells, the different NTDs of these proteins, which include the activation function 1 (AF1) domain, one of the two transcriptional activation domains in these receptors, partially destabilise the proteins through a ubiquitination-dependent mechanism [48]. Although these experiments were performed in a heterologous system and did not reveal similar regulation for the EcR-B2 isoform, which has a much shorter AF1 domain, we tested whether the NTD plays a role in SC-specific, BMP-dependent control of EcR protein levels. We expressed EcR-C, an artificial isoform of the protein in which the NTD sequence has been deleted, in SCs. This protein only contains sequences common to all isoforms [49], and in other cell types, it usually has reduced activity compared with native forms of EcR when overexpressed.

**Fig 7:**
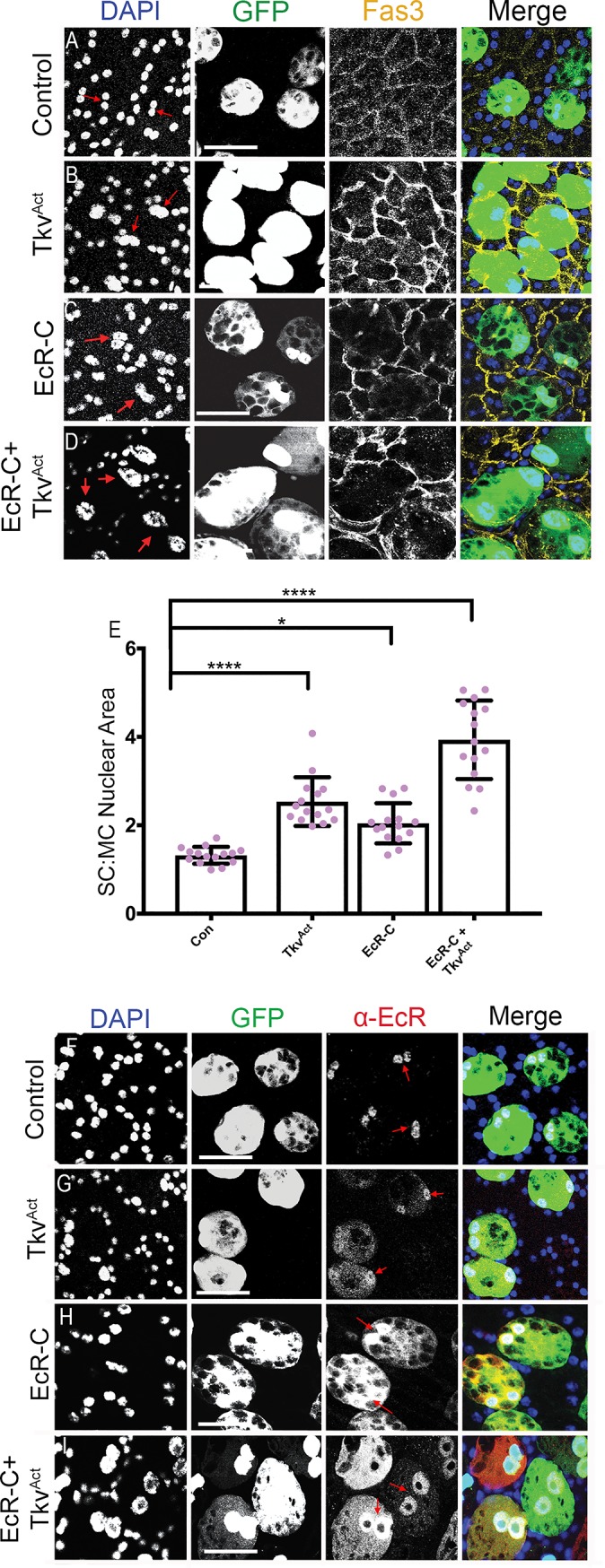
Sequences associated with the AF1-containing N-terminal domain of different EcR isoforms are involved in BMP-dependent regulation. Dissected AGs from 6-day-old virgin males expressing GFP and other transgenes under esg^ts^F/O control were stained either with an antibody against Fas3 to mark the apical outlines of SCs and neighbouring MCs (yellow; [A-D]) or with a pan-EcR antibody (red arrows; [F-I]) and DAPI (blue nuclei). (A-D) EcR-C overexpression promotes SC nuclear growth (C) relative to controls (A). Combining EcR-C overexpression with elevated BMP signalling induced by Tkv^ACT^ synergistically drives further SC nuclear growth (D) significantly greater than Tkv^ACT^ alone (B). (E) Histogram showing the size of SC nuclei relative to MC nuclei in AGs in which SCs express different transgenes. (F-I) Overexpression of the N-terminally truncated EcR protein, EcR-C, leads to accumulation of nuclear and cytosolic EcR (H) compared with controls (F). Coexpression with Tkv^ACT^ appears to increase the ratio of nuclear to cytosolic EcR in some cells (I) and lead to much higher levels of EcR protein in SCs than with Tkv^ACT^ alone (G). Significance was assessed by one-way ANOVA and Tukey’s multiple-comparisons test. *p < 0.05, ****p < 0.0001, *n* ≥ 15. Scale bars, 50 μm. Underlying data for this figure can be found in S1 Data. AF1, activation function 1; AG, accessory gland; BMP, bone morphogenetic protein; Con, control; EcR, ecdysone receptor; *esg*, *escargot*; esg^ts^F/O, the yeast transcription factor GAL4 expressed under the control of the promoter of the gene *esg* in a temperature-dependent fashion; Fas3, Fasciclin3; GFP, green fluorescent protein; MC, main cell; SC, secondary cell; Tkv, Thick veins.

Unlike the endogenously expressed isoforms of EcR, overexpression of EcR-C promoted growth of SCs (Fig 7A, 7C and 7E, S1 Data) and produced high levels of EcR protein in the nuclei and cytosol of SCs when expressed alone (Fig 7F and 7H). A simple explanation of our data, given the known role of EcR NTD sequences in protein stability [48], is that BMP signalling activity regulates levels of different EcR isoforms via a genetic interaction with each of their unique NTDs. Alternatively, BMP signalling may affect transcript levels specifically via the sequences encoding each NTD, which are absent in the *EcR-C* transcript. The growth effects of EcR-C were further enhanced by coexpression with Tkv^ACT^ in virgin males (Fig 7C–7E), indicating that BMP signalling affects EcR activity via more than one mechanism. Indeed, relative levels of nuclear versus cytosolic EcR-C appeared to be increased by Tkv^ACT^ overexpression in many, but not all, SCs, suggesting that BMP signalling might also regulate the nuclear trafficking of EcR (Fig 7G–7I).

Regulation of the EcR by BMP signalling that is independent of transcription initiation has not previously been reported in *Drosophila*. It was not observed in MCs (S4I and S4J Fig). We conclude that BMP signalling controls EcR levels and EcR signalling in SCs via a cell type–specific interaction that appears to partly involve the NTD of each EcR isoform or the transcript sequences encoding these NTDs.

### BMP and EcR signalling function antagonistically to regulate SC migration

We have previously shown that BMP signalling promotes not only SC growth [9] and secretion [14] but also migratory activity of SCs. A small subset of SCs delaminates apically from the AG epithelium after multiple matings and migrates to the proximal end of the gland [9], where the cells can be transferred to females following further mating. Activating BMP signalling in adult SCs by expressing Tkv^ACT^ induces a similar phenotype in virgin males. We sought to test whether EcR plays a role in this migration. Whereas increased BMP signalling activity promoted spontaneous delamination and migration of SCs in virgin males (S5A, S5B, and S5H Fig, S1 Data), coexpression with EcR isoforms markedly reduced the number of migrating cells (S5D–S5H Fig). In fact, knocking down *EcR* transcripts in virgin males also appeared to promote low levels of SC migration (S5C and S5H Fig, S1 Data). This suggests that, in contrast to growth regulation, the EcR acts antagonistically to BMP signalling in migration control.

### Mating drives synthesis of new DNA in SCs, which is regulated by BMP-dependent, but hormone-independent, EcR signalling

**Fig 8:**
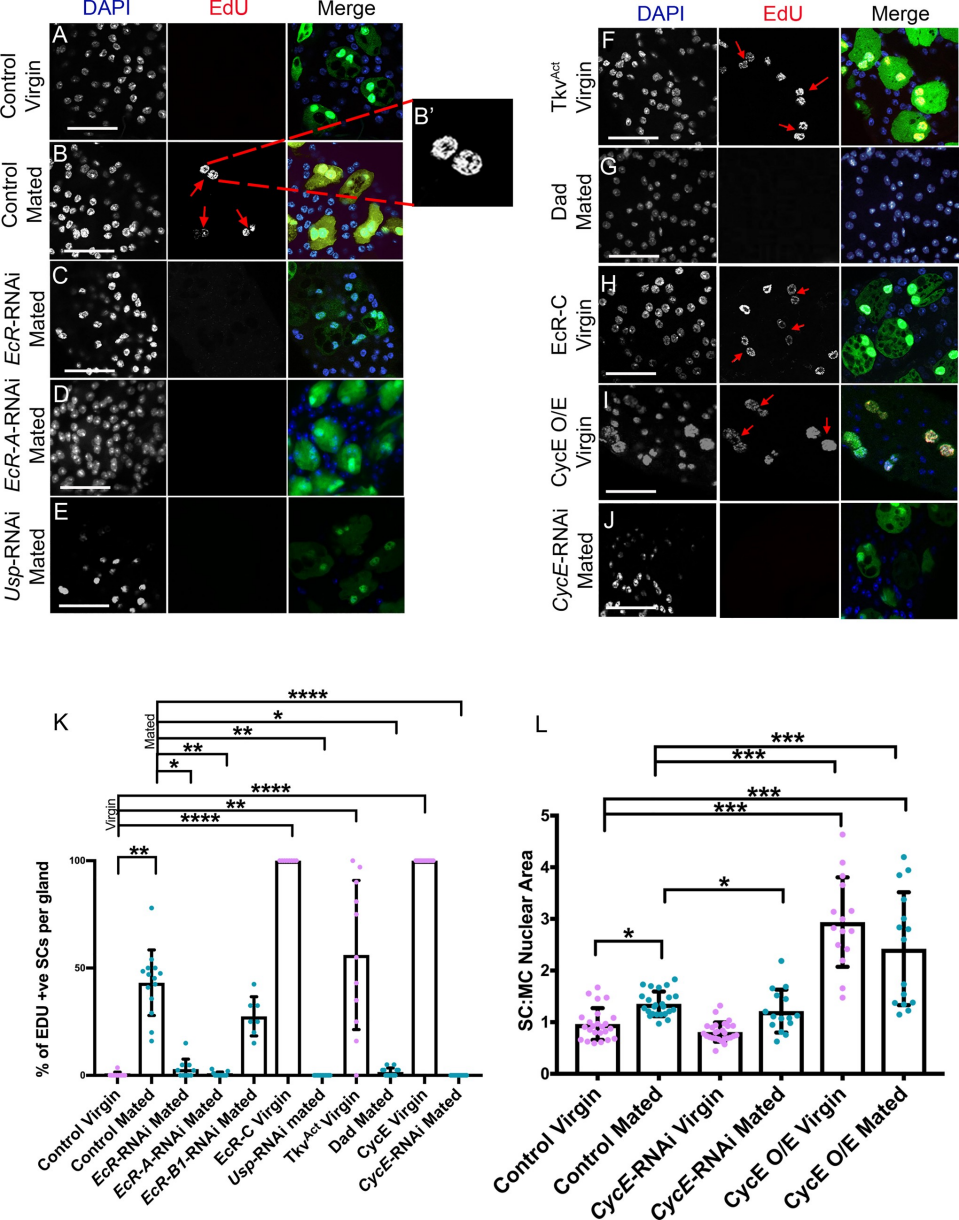
EcR-mediated endoreplication of SC DNA is stimulated by mating. Males expressing GFP and other transgenes under esg^ts^F/O control were cultured on EdU-containing food posteclosion and dissected at 6 days, and their AGs were probed for EdU uptake to assess adult DNA replication and stained with DAPI. (A, B) EdU is incorporated in about 30%–40% of adult SCs after mating ([B]; arrows depict SCs with EdU uptake) but not in virgins (A). Enlargement in (B′) shows a high-magnification view of a single SC. (C-E) SC-specific expression of *EcR*-RNAi (C), *EcR-A*-RNAi (D), and *Usp*-RNAi (E) blocks EdU incorporation in mated males. (F-H) Expression of Tkv^ACT^ (F) or EcR-C (H) in SCs promotes EdU incorporation in SCs from virgin males, whereas Dad-expressing SCs do not incorporate EdU after mating ([G]; note that weak GFP expression in these cells is masked following the EdU staining procedure). (I, J) Overexpression of *CycE* in SCs of virgin males induces endoreplication (I), whereas knockdown of *CycE* in mated males suppresses it (J). (K) Histogram showing EdU incorporation into SC nuclei in different genetic backgrounds with and without mating. (L) Histogram showing SC nuclear size relative to adjacent MC nuclei in 6-day-old males for control glands and glands expressing different transgenes affecting CycE levels in SCs under esg^ts^F/O control. Data were analysed by the Kruskal-Wallis test with Dunn’s multiple-comparison test (K) or by one-way ANOVA with Dunnett’s multiple-comparisons test (L). *p < 0.05, **p < 0.01, ***p < 0.001, ****p < 0.0001, *n* ≥ 10 (K), *n* ≥ 15 (L). Scale bars, 70 μm. Underlying data for this figure can be found in S1 Data. +ve, positive; AG, accessory gland; *CycE*, Cyclin E; Dad, Daughters against Decapentaplegic; EcR, ecdysone receptor; EdU, 5-ethynyl-2′-deoxyuridine; *esg*, *escargot*; esg^ts^F/O, the yeast transcription factor GAL4 expressed under the control of the promoter of the gene *esg* in a temperature-dependent fashion; GFP, green fluorescent protein; O/E, overexpression; RNAi, RNA interference; SC, secondary cell; Tkv, Thick veins; *Usp*, Ultraspiracle.

In *Drosophila*, increased nuclear and cell size is often associated with endoreplication, which increases gene expression through elevated gene copy number [50]. At eclosion, both SCs and MCs have two large nuclei, each estimated to be tetraploid, because of endoreplication during pupal development [51]. Previously we were unable to detect additional endoreplication in adult SCs of mated flies fed with the nucleotide analogue bromodeoxyuridine (BrdU) [9]. However, we reasoned that this might be explained by poor penetration of the anti-BrdU antibody in AGs, and we therefore repeated these experiments by feeding males during adulthood with 5-ethynyl-2′-deoxyuridine (EdU), which can be detected chemically. Although EdU uptake was rarely observed in SCs of 6-day-old virgin males, approximately 30%–40% of SCs from multiply-mated males incorporated EdU, indicating that new DNA synthesis was occurring in a subset of these adult cells (Fig 8A, 8B and 8K, S1 Data). No new DNA synthesis was observed in the MCs of either virgin or mated glands (Fig 8A and 8B).

**Fig 9:**
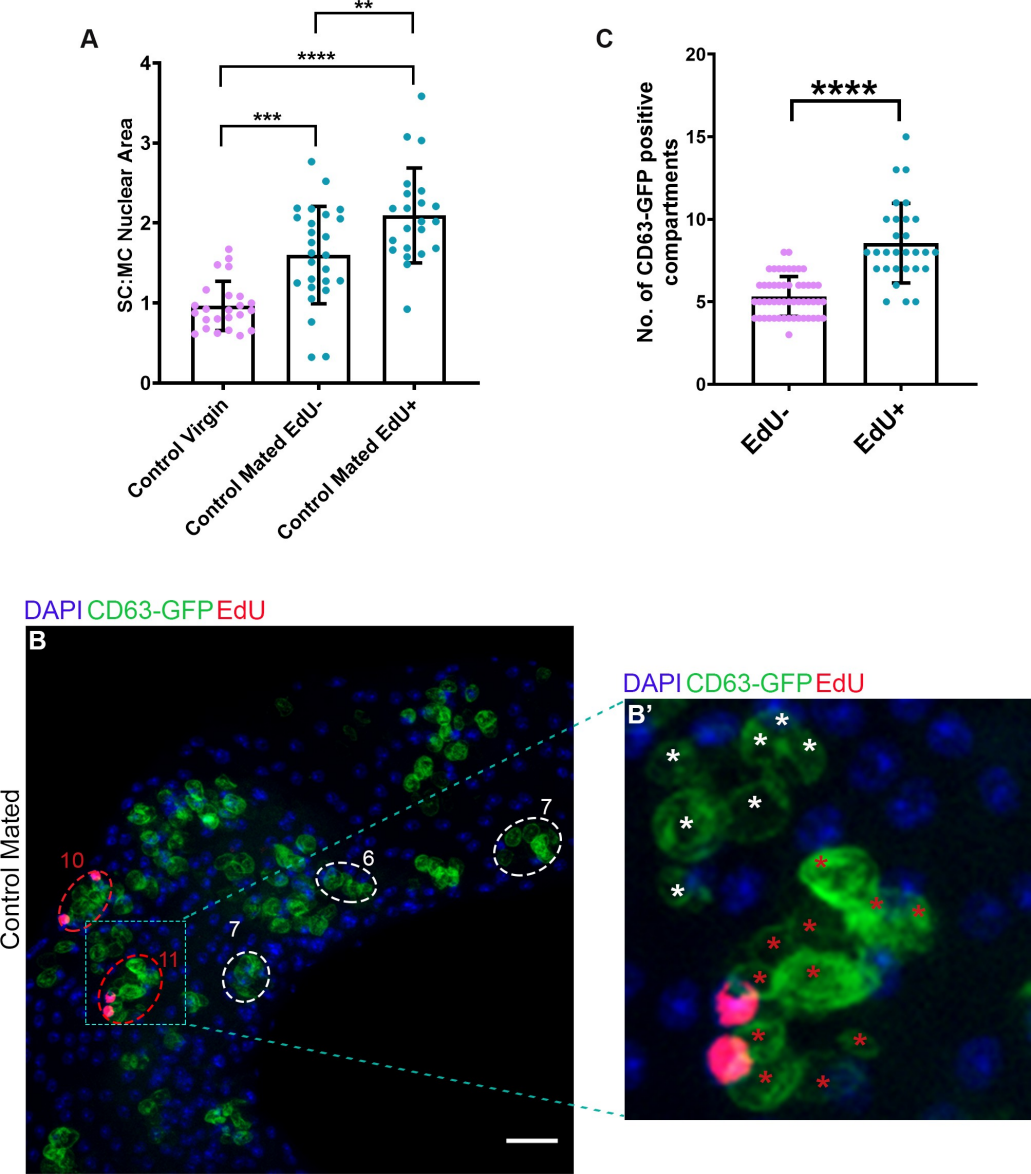
Endoreplicating SCs have larger nuclei and greater biosynthetic secretory activity than other SCs. (A) Histogram showing SC:MC nuclear size ratio for EdU-positive and EdU-negative SCs from esg^ts^F/O control mated males versus virgins. (B) Confocal microscopy image of males after a 24-hour pulse of CD63-GFP expression in SCs directly after multiple matings. Some SCs are marked by dashed circles (red for SCs with adult endoreplication, white for those without; number of CD63-GFP-marked compartments is given in each case). Not all labelled compartments can be seen in this single z-plane. Magnified maximum projection image shows examples of one EdU-positive and one EdU-negative SC, with each labelled compartment marked with a red or white asterisk, respectively. (C) Histogram showing number of CD63-GFP-positive compartments per cell in EdU-positive and EdU-negative SCs from multiply-mated males, following a 24-hour pulse of CD63-GFP expression. Data analysed by one-way ANOVA with Sidak’s multiple-comparisons test (A) or a two-tailed Mann-Whitney test (C). **p < 0.01, ***p < 0.001, ****p < 0.0001, n ≥ 21 (A), n ≥ 29 (C). Scale bars, 20 μm. Underlying data for this figure can be found in S1 Data. EdU, 5-ethynyl-2′-deoxyuridine; *esg*, *escargot*; esg^ts^F/O, the yeast transcription factor GAL4 expressed under the control of the promoter of the gene *esg* in a temperature-dependent fashion; GFP, green fluorescent protein; MC, main cell; No., number; SC, secondary cell.

Interestingly, SC nuclei that take up EdU in mated glands were larger than EdU-negative nuclei (Fig 9A, S1 Data), indicating that part of the increase in SC nuclear size is a consequence of new DNA synthesis. Furthermore, EdU incorporation was distributed across all parts of the nucleus (Fig 8B′), suggesting that it is not the result of focal gene amplification, as is seen for chorion genes in ovarian follicle cell nuclei [52].

We have previously shown that SC secretion of exosomes is under BMP control [14] and that mating induces increased secretory activity in SCs [13]. To test whether endoreplication in adult SCs drives enhanced biosynthetic activity in the secretory system compared with other SCs, we expressed a green fluorescent protein (GFP)-tagged form of the human exosome marker CD63-GFP in a 24-hour pulse in multiply-mated males [14]. This protein labels all large secretory compartments in SCs, and therefore, the number of GFP-positive compartments produced during the pulse of expression provides a measure of the rate of compartment biogenesis, which is approximately one compartment per 4 or 5 hours in virgin males [13,14]. There were significantly more labelled compartments in SCs that had undergone endoreplication than those that had not (Fig 9B and 9C, S1 Data), consistent with the hypothesis that a key role for endoreplication in adults is to increase secretory biosynthetic capacity.

To further assess whether genome endoreplication is responsible for mating-dependent growth, we tested the effect of overexpressing and knocking down the G1/S cyclin, Cyclin E (CycE), in adult SCs. CycE is required for endoreplication in other *Drosophila* cell types [50]. Although knockdown of *CycE* had no significant effect on nuclear growth in virgin males, it inhibited endoreplication and additional SC nuclear growth in mated males (Fig 8J–8L). Furthermore, overexpression of *CycE* in virgin males stimulated nuclear growth and endoreplication in all adult SCs, but this growth was not enhanced further by mating (Fig 8I, 8K and 8L, S1 Data). Consistent with these findings, studies of endoreplication in the salivary gland have suggested that constant overexpression of CycE can drive one cycle of endoreplication but does not permit further rounds [53].

Because both BMP and EcR signalling modulate SC nuclear growth in mated males, we tested whether these pathways regulate DNA synthesis in adult SCs. In complete contrast to controls, the majority of SCs expressing Tkv^ACT^ in adult virgin males typically incorporated EdU over 6 days (Fig 8F and 8K, S1 Data). Furthermore, all SCs expressing the EcR-C construct contained nuclear EdU (Fig 8H and 8K, S1 Data). EdU uptake was significantly suppressed in glands from multiply-mated males expressing EcR-RNAi or the BMP antagonist Dad in SCs (Fig 8C, 8G and 8K, S1 Data). Consistent with their effects on SC nuclear growth after mating, *EcR-A*-RNAi, but not *EcR-B1*-RNAi, completely suppressed endoreplication (Fig 8D and 8K, S1 Data), demonstrating that EcR-A plays the critical role in this process.

**Fig 10:**
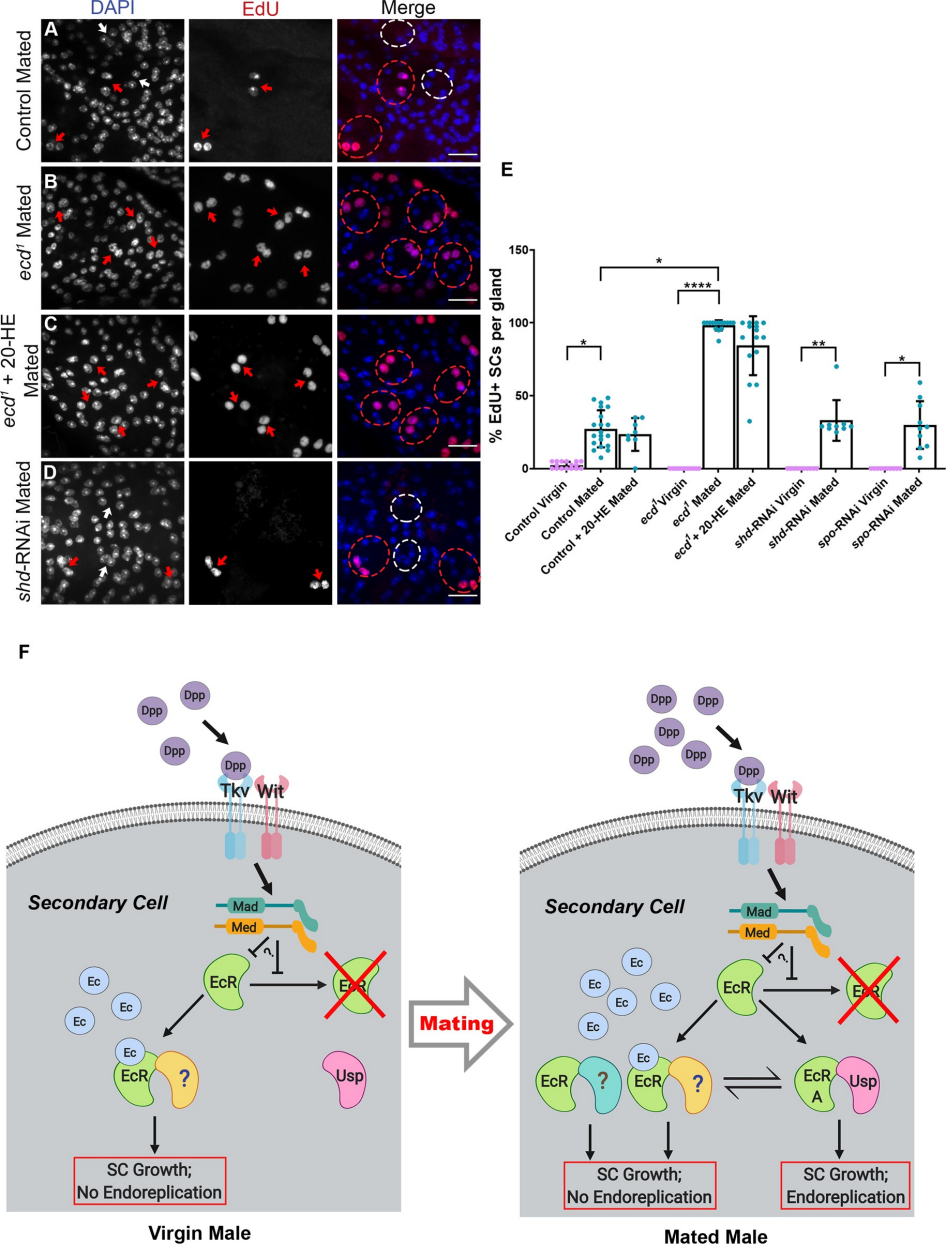
Hormone-independent, EcR-mediated endoreplication of SC DNA is stimulated by mating. Males expressing GFP and other transgenes under esg^ts^F/O control or *ecd*^*1*^ mutants were cultured on EdU-containing food posteclosion and dissected at 6 days, and their AGs were probed for EdU uptake to assess DNA replication and stained with DAPI. SCs were recognised by GFP expression or by their characteristic vacuolar morphology in *ecd*^*1*^ mutants; selected cells are highlighted by dashed circles (red for EdU-positive, white for EdU-negative). (A) EdU is incorporated in about 30%–40% of SCs after mating (red arrows depict SCs with EdU uptake, white arrows mark those without EdU). (B, C) Almost all SCs in *ecd*^*1*^ males contain EdU in their nuclei after mating (B), and this is unaffected by 20-HE feeding (C). (D) SC-specific expression of *shd*-RNAi has no effect on the number of SCs containing EdU in mated males. (E) Histogram showing EdU incorporation into SC nuclei in different genetic backgrounds. (F) Proposed model explaining the different forms of hormone-dependent and hormone-independent, EcR-mediated SC growth in virgin and mated males. Our data reveal that EcR-dependent growth of SCs is differentially modulated by the presence of Ec according to mating status. An Ec-EcR complex, which requires a dimerisation partner other than USP (marked ?; this could be an EcR dimer, for example) is necessary for normal SC nuclear growth observed in virgin males. In mated males, SC growth is enhanced, at least in part due to new DNA synthesis driven by a hormone-independent EcR-A/USP complex. This complex must either repress or activate a subset of genes that are not EcR targets in virgin males, hence inducing cell cycle regulators like *CycE* either directly or indirectly. In mated males, EcR complexes that do not involve USP can also drive non-endoreplication-mediated growth, potentially via hormone-dependent and hormone-independent mechanisms, which may be coordinately regulated with endoreplication (e.g., this growth appears to increase following *Usp* knockdown). EcR levels and signalling are stimulated by elevated BMP signalling induced by autocrine BMP ligand Dpp through the heterodimeric Tkv/Wit receptor [13]. Data were analysed by the Kruskal-Wallis test with Dunn’s multiple-comparisons test. *p < 0.05, **p < 0.01, ****p < 0.0001, n ≥ 8. Scale bars, 20 μm. Underlying data for this figure can be found in S1 Data. 20-HE, 20-hydroxyecdysone; AG, accessory gland; BMP, bone morphogenetic protein; *CycE*, *Cyclin E*; Dpp, Decapentaplegic; Ec, ecdysone; EcR, Ec receptor; *ecd*, *ecdysoneless*; EdU, 5-ethynyl-2′-deoxyuridine; *esg*, *escargot*; esg^ts^F/O, the yeast transcription factor GAL4 expressed under the control of the promoter of the gene *esg* in a temperature-dependent fashion; GFP, green fluorescent protein; Med, Medea; RNAi, RNA interference; SC, secondary cell; *shd*, *shade*; *spo*, *spook*; Tkv, Thick veins; USP, Ultraspiracle, Wit, Wishful thinking.

Given the surprising finding that mating-dependent, EcR-mediated growth is hormone independent, we explored whether blocking ecdysone synthesis had any effect on endoreplication. The number of SCs incorporating EdU in *ecd*^*1*^ males shifted to the nonpermissive temperature after eclosion was assessed in virgin and mated males. In contrast to virgins, in which no endoreplication was observed, incorporation in multiply-mated males was increased compared with controls, with positive staining in virtually all SCs, suggesting that mating-induced, EcR-mediated endoreplication is not hormone dependent (Fig 10A, 10B and 10E, S1 Data). However, levels of endoreplication were also elevated when mated *ecd*^*1*^ males were fed with 20-HE (Fig 10C and 10E, S1 Data), indicating that the increase in endoreplication in this mutant, when mated, might not be due to changes in 20-HE. Importantly, though, in support of a hormone-independent control of endoreplication, knockdown of either *spo* or *shd* in SCs also had no effect on the proportion of endoreplicating SCs after mating (Fig 10D and 10E, S1 Data). Taken together, these data indicate that both BMP and EcR signalling cooperate in SCs of mated males to promote synthesis of new DNA. Endoreplication explains much of the additional nuclear growth in a subset of SCs after mating (Fig 9A, S1 Data), but unlike growth in virgin males, this process is ecdysone independent.

Because the form of EcR signalling that controls mating-dependent growth differs from virgins, we tested whether it might involve USP, the well-characterised transcriptional partner of EcR. SC-specific *Usp* knockdown in mated males blocked endoreplication (Fig 8E and 8K, S1 Data), mirroring *EcR-A* knockdown, demonstrating that this aspect of hormone-independent growth is likely to be controlled by an EcR-A/USP heterodimer. However, *Usp* knockdown did not suppress the additional growth in SCs associated with mating (Fig 3I, S1 Data), suggesting that mating-dependent, endoreplication-independent growth does not require USP and, indeed, that it may be up-regulated in the absence of this molecule.

## Discussion

Secretory cells in both the mammalian prostate and the fly AG have unusual growth properties in adults. Androgens play a central role in regulating growth and proliferation in the prostate, potentially linking nutrition and sexual activity to adult glandular function [22,23], as well as driving maturation during puberty. In advanced prostate cancer, when tumour cells can become resistant to antiandrogen treatment, they frequently grow via a hormone-independent, AR-driven process that remains incompletely understood [24].

Here, we demonstrate that SC nuclear growth in virgin male flies also involves hormone-dependent steroid receptor activity. This activity is modulated by local autocrine BMP signals via a novel mechanism. After mating, EcR-dependent nuclear growth becomes partially hormone independent and requires CycE-driven endoreplication. As we discuss below, these regulatory mechanisms drive increased SC growth and secretion after mating, independently of ecdysone concentration, so that SC products that have been transferred to females can be rapidly replenished in males.

### BMP signalling tightly regulates EcR levels to control SC growth

Our findings that BMP signalling is required in SCs for them to express detectable levels of the EcR and that knocking down EcR expression blocks BMP-stimulated growth strongly indicate that EcR signalling is a primary mediator of BMP-dependent growth-regulatory effects in these cells. The most abundant EcR isoform, EcR-B1, plays an important in this process in virgin males, but as in the developing AG [34], it is clear that EcR-A is also involved in SC growth and can be regulated by BMP signalling (S1 and S2 Figs), despite being expressed at lower levels.

The BMP/EcR interaction, which does not involve transcriptional control of *EcR* expression, appears highly cell type specific in flies. It cannot be induced in MCs of the AG and has not been reported in multiple tissue types during *Drosophila* development. Signalling by Activins, members of another class of transforming growth factor β (TGF-β) ligands, is required for EcR-B1 expression during neuronal remodelling of the fly brain at metamorphosis [54]. However, the effects of losing the type I Activin receptor Baboon can be overcome by GAL4/UAS-driven expression of EcR-B1, suggesting a direct transcriptional mechanism [54]. Regulation of EcR-B1 expression by TGF-β/BMP signalling has also been reported in larval motoneurons as they dismantle during metamorphosis, but the detailed mechanisms involved have not been characterised [55].

Each of the three normal isoforms of EcR has a unique NTD, which includes a so-called AF1 transcriptional activation domain. The EcR-C protein lacks an NTD, and it can partially evade control by BMP signalling. The transcript sequences encoding the normal EcR isoforms and EcR-C are all identical, except in the 5′ regions that encode the isoform-specific NTDs. Either these different 5′ sequences are all independently targeted by a mechanism that mediates BMP-dependent control of EcR protein levels or the regulation is posttranslational. The latter appears more likely because the NTDs of both EcR-A and EcR-B1 are involved in degradative mechanisms that control EcR protein levels [48]. However, because we have not been able to assess *EcR* transcript levels in SCs in different genetic backgrounds, we cannot exclude a transcript-specific regulatory mechanism.

### Multiple forms of noncanonical EcR signalling in SCs control growth in virgin and mated males

Not only is the EcR regulated via a unique BMP-dependent mechanism in SCs, it also has an unusual and complex mode of action and target specificity (Fig 10F). Although USP, the well-characterised binding partner of EcR during development, is expressed selectively in the nuclei of SCs, *Usp* knockdown does not alter SC nuclear size in either virgin or mated males. In virgin males, knockdown of either *EcR-B1*, which is abundantly expressed in SCs, or *EcR-A* suppresses SC nuclear growth. We were unable to detect any role for EcR-B2 using a dominant negative construct.

USP-independent EcR signalling has been reported previously in one developmental scenario in larvae [56]; the mechanisms involved have not been characterised, although the authors propose that the EcR may act as a homodimer or bind with an alternative partner. Furthermore, recent work has shown that expression of multiple EcR isoforms is functionally important in development of the adult AG epithelium but that USP is not required [34]. These authors did not identify the cells involved or the precise cellular defects.

We have screened for expression of several of the known target genes of EcR in development, such as *Broad* and *Ecdysone-induced protein 74EF* (*Eip74EF*), using well-characterised antibodies [57] and specific gene traps but have not been able to identify any downstream targets of the EcR in SCs. Cell type–specific analysis of genomic EcR binding sites or the SC transcriptome will be required to unravel the genetic programme controlled by the specialised EcR signalling taking place in these cells.

Whereas EcR signalling in SCs of virgin males is potentially mediated by a single hormone-dependent mechanism, multiple mechanisms are involved in mated males (Fig 10F). One of these mechanisms appears to involve an EcR-A/USP heterodimer and produces a mating-specific and hormone-independent downstream effect, endoreplication. All three genetic manipulations that reduce ecdysone levels and inhibit SC nuclear growth in virgin animals (the *ecd*^*1*^ mutation and *shd*/*spo* knockdown) did not suppress growth or endoreplication in mated males. In fact, in the *ecd*^*1*^ mutant, endoreplication occurred in almost all SCs, although this might be the result of an ecdysone-unrelated effect of the mutant [40] because it was not suppressed by 20-HE feeding.

Epithelial cells in the AG endoreplicate during pupal development [51], and this process is essential for normal growth of the gland [58]. However, in adults, we find that endoreplication is normally only seen upon mating (Fig 8) and is responsible for much of the additional SC growth seen after mating (Fig 9A, S1 Data). Indeed, *CycE* knockdown specifically suppresses mating-dependent growth. Endoreplication and increased growth can be phenocopied in virgin males by activation of BMP signalling or overexpression of EcR-C in SCs. Expressing any EcR isoform in SCs with elevated BMP signalling induces excessive levels of nuclear growth (Fig 5 and S1 Fig). However, it remains unclear whether these effects mirror the normal EcR-dependent mechanism controlling endoreplication after mating or a novel form of regulation induced by the artificially high EcR levels in these genetic backgrounds.

Hyperactivated BMP signalling may be an important trigger for endoreplication after mating. We have previously shown that only about 30% of SCs increase BMP signalling detectably following multiple matings [13], mirroring the proportion of endoreplicating cells under these conditions. However, we have yet to develop a robust protocol with which we can codetect EdU and the BMP transcriptional target, phosphorylated Mothers against Decapentaplegic (P-Mad), to confirm that these two populations are the same. Furthermore, because genetically activating BMP signalling in SCs does not induce endoreplication in all cells, there is probably a second as-yet-unidentified mechanism that modulates the number of endoreplicating cells.

Because SCs in mated males, which have not endoreplicated, are larger than in virgin males (Fig 9A, S1 Data), the EcR must also regulate other forms of mating-dependent nuclear growth that are not linked to endoreplication. We have not characterised these in detail (Fig 10F). However, as in virgin males, this growth potentially reflects greater decondensation of chromatin. Interestingly, SC-specific *Usp* knockdown blocks endoreplication but not mating-dependent growth (Figs 8E, 8K and 3I, S1 Data), suggesting that endoreplication-independent growth mechanisms can sometimes compensate when endoreplication-dependent growth is inhibited. Because mating-dependent growth does not appear to be suppressed by genetic manipulations that reduce ecdysone levels (Fig 3J, S1 Data), it seems likely that the endoreplication-independent form of this EcR-mediated growth is at least partly hormone-independent. However, we cannot exclude the possibility that hormone-dependent growth is also involved and that the balance of hormone and EcR isoform dependency varies depending on ecdysone levels and genetic background.

Finally, although BMP and EcR signalling cooperate in controlling growth, in both virgin and adult males, their effects appear to be antagonistic in the regulation of cell migration. Presumably BMP’s promigratory functions are mediated via non-EcR-dependent pathways, which are typically dominant to the EcR in those SCs that normally delaminate from the AG epithelium after multiple matings [9].

### Functional consequences of mating-dependent, hormone-independent endoreplication

As well as promoting SC growth, we found that adult endoreplication increases the rate of secretory compartment formation in mated males (Fig 9). Endoreplication is employed to promote high levels of transcriptional activity and secretion in a range of organisms from mammals to plants [50]; for example, in the salivary glands and follicle cells of the egg chamber in *Drosophila*. In SCs, up-regulation of gene expression following genomic duplication and increased secretory activity presumably facilitates the rapid replenishment of secreted SC products that are extruded from the AG as a result of mating.

So why might adult SC endoreplication and other EcR-mediated, mating-induced growth be hormone-independent? Other studies have shown that whole-animal 20-HE titres increase in male flies exposed to previously mated females [27] and that EcR signalling activity in the fly brain is required for normal courtship behaviours [27,29]. The functional significance of reducing ecdysone levels and SC growth in virgin males, particularly in the absence of females, is likely to involve the conservation of resources. A hormone-independent endoreplication mechanism allows such female-deprived flies to rapidly up-regulate SC activity following mating using the EcR.

Unfortunately, the experimental conditions under which 20-HE has been shown to be altered in response to female rejection [27] and sleep deprivation [59] involve short-term changes, which are very unlikely to affect SC nuclear growth over the 6-day period that we study. When we extended the ‘rejection regime’ for 6 days, we were unable to detect significant changes in 20-HE levels and consequently saw no effects on SC nuclear size. Other treatments that induce long-term physiological changes in endogenous 20-HE levels will need to be defined in virgin males before we are able to test the hypothesis that EcR-regulated growth plays an important role in conserving resources under different social or environmental conditions.

Could the ecdysone-dependent control of SC activity have physiological functions outside of SCs? Application of topical 20-HE to males exposed to females, which have an experimentally sealed ovipositor that prevents mating, rescues the reduction in AG secretory activity exhibited by these animals [33], strongly suggesting that the overall activity of the gland can be influenced by this hormone. We propose that direct effects of ecdysone on SCs, cells which have an important role in AG reproductive function [9–11] and can affect the normal processing of MC products like Ovulin [11], provide one route by which this hormone can alter the activity of the entire gland. Indeed, Sitnik and colleagues [60] have presented evidence that blocking the normal development of SCs in a specific *Abdominal B* (*Abd-B*) mutant may have indirect effects on the transcriptional programme of MCs, further supporting the idea that SCs can coordinate functions of both epithelial cell types in the AG.

### Growth in both prostate cells and fly SCs is regulated by BMPs and different forms of steroid receptor signalling

Previous work has suggested several similarities between mammalian prostate cells and *Drosophila* SCs, including cell growth in adults [9] and the secretion of exosomes that can fuse with sperm when transferred to females during mating [14]. We initially tested the function of EcR signalling in SCs because of the critical role of androgens and AR signalling in normal and tumorigenic prostate epithelial growth. This has uncovered further potential similarities between humans and flies: the receptor for a steroid hormone, which is regulated by sociosexual experience and environment, is involved in controlling growth of secretory cells in both male glands and can function by hormone-dependent and hormone-independent mechanisms. In flies, the switch to hormone independence plays a physiological role, whereas in the prostate, it has only been observed to date in cancer. Furthermore, in both organisms, cells make active steroid hormone locally, promoting an autocrine form of hormone-dependent growth signalling.

Of course, there are also significant differences between these steroid receptor–regulated forms of growth in humans and flies. The human prostate grows by hyperplasia, whereas SCs are hypertrophic postmitotic cells. Nevertheless, the existence of hormone-independent steroid receptor signalling in advanced prostate cancer cells and in SCs is a surprising finding given that hormone-independent EcR signalling has not been observed in any other *Drosophila* cell type. Furthermore, this form of EcR signalling partly acts through the control of cell cycle genes, though this is not coupled to nuclear and cellular division as it is in the prostate. Inevitably, there will be some genetic changes associated with the development of prostate cancer that are not involved in normal SC biology. However, it will be interesting to test whether other cell cycle regulators, which are specifically associated with castration-resistant prostate cancer [61,62], also play a role in hormone-independent signalling in SCs.

Like steroids, BMP signalling has been implicated in prostate growth and metastasis [15–17]. However, its effects are complex because different BMP ligands, which signal through alternative pathways, can have opposite effects. In prostate cancer, BMP signalling has been implicated in the androgen-independent AR signalling associated with castration resistance [63–65], though its mode of action remains unclear. Our findings suggest links between BMPs and steroid receptors in the male reproductive system of the fly that switch the EcR to a hormone-independent mode under physiological conditions. The mechanisms involved now require further investigation in both flies and humans, with particular focus on defining the cellular conditions under which BMP-induced, hormone-independent signalling is activated.

## Materials and methods

### Fly strains and culture

The following fly strains (obtained from the Bloomington Stock Centre, except where noted) were employed: esg^ts^F/O (*w; esg-GAL4*, *UAS-GFPnls; act>CD2>GAL4*, *UAS-FLP*; gift from B. Edgar) [35], *UAS-EcR-RNAi* (TRiP.JF02538) [66], *UAS-EcR-B1*, *UAS-EcR-A*, *UAS-EcR-B2*, *UAS-EcR-C*, *UAS-EcR-B2-ΔC655*.*W650A* [49,67], *UAS-EcR-B1-RNAi*, *UAS-EcR-A-RNAi* [38], *UAS-Tkv*^*Q199D*^ [47], *UAS-Med-RNAi* [68], *UAS-Usp-RNAi* (TRiP.JF02546), *UAS-ry-RNAi* (TRiP.44106), *UAS-CycE-RNAi* (TRiP.GL00511), *UAS-CycE* [69], *UAS-CD63-GFP* [14] (gift from S. Eaton), *dsx-GAL4* [14], and *Acp26Aa-GAL*4 (gifts from S. Goodwin) [70]. TRiP UAS-RNAi lines are described in [71]. Flies were fed on standard cornmeal agar medium. No dried yeast was added to the vials. 20-HE-containing food was prepared by mixing standard medium with a 10 mM 20-HE (Selleckchem/Cambridge Biosciences) stock solution to a final concentration of 0.1 mM with or without 0.2 mM EdU (see below). Flies were cultured on this food from eclosion until analysis at 6 days.

### Fly genetics

To express UAS-transgenes in adult SCs under esg^ts^F/O control [9], fly crosses were initially cultured at a nonpermissive temperature, 18°C or 25°C (*w*^*1118*^ and *UAS-ry-RNAi* flies were used in control crosses with the esg^ts^F/O strain). Newly eclosed virgin males of the appropriate genotype were selected, separated from females, and transferred to 28.5°C immediately. All SCs that induce FLP-mediated recombination of the *act>CD2>GAL4* construct continue to express GFP. Mosaic experiments were performed by delaying the temperature shift until day 3 of adult life, with dissection of adults at approximately 9 days posteclosion [9]. Expression of *esg-GAL4* is gradually lost in some adult SCs, so delaying the temperature shift results in *act>CD2>GAL4* recombination in a subset of SCs. Combining the dominant negative EcR constructs with the esg^ts^F/O line produced very few adults of the appropriate genotype, presumably due to leaky expression during development. For the EcR-B2 dominant negative experiment, SC-specific *dsx-GAL4* under GAL80^ts^ regulation [14] was therefore employed as an alternative driver. For nuclear size measurements and growth analysis, males were typically dissected at 6 days.

### Male rejection regime

Virgin males were isolated in vials shortly after eclosion. Each male was placed overnight with a white-eyed premated female, and this was repeated daily until day 6. Premated females had been mated the previous day by incubating with three virgin white-eyed males; the vials were observed to ensure mating occurred. Virgin females were kept after mating, and progeny were checked for eye colour to ensure the ‘rejected’ males (which were all red eyed) had not successfully mated. At 6 days, the rejected males were dissected and prepared for immunostaining or used for 20-HE measurements.

### Measurement of whole-body 20-HE

Ecdysteroid levels were measured using an enzyme immunoassay (EIA) 20-HE EIA SPI-Bio kit (A05120; Cayman Chemicals); 20-HE and 20-E acetylcholinesterase were used as the standard and enzymatic tracer, respectively [27]. For sample preparation, 10 flies per condition were homogenised in ice-cold PBS and centrifuged at 10,000*g* for 15 minutes. To extract 20-HE, the precipitates were resuspended in 1 ml methanol and centrifuged at 10,000*g* for 10 minutes. The supernatants were then evaporated to dryness and resuspended in 100 μl of EIA buffer. Ellman’s reagent was used for the chromogenic reaction, and absorbance was read at 415 nm using a Victor3 multilabel plate reader (Perkin Elmer). All assays were performed in triplicate.

### Immunohistochemistry and imaging

This followed previously published methods [9,14]. Flies were anaesthetised using CO_2_ and dissected with fine forceps in 4% paraformaldehyde dissolved in PBS. Dissected AGs were transferred to Eppendorf tubes, fixed for 20 min at 22°C, and then washed 6 × 10 minutes in 1 ml PBST (1× PBS, 0.3% Triton X-100 [Sigma-Aldrich]). Anti-Fas3 [72], anti-pan-EcR, anti-EcR-A, and anti-EcR-B1 [36] antibodies were all obtained as supernatants from the Developmental Studies Hybridoma Bank, Iowa, and diluted 1 in 10 in PBSTG (PBST, 10% goat serum). Mouse anti-USP antibody (1:100 dilution) was a kind gift from the Kafatos laboratory [46], and the rabbit anti-ANCE antibody (1 in 200) was kindly provided by E. Isaac [73]. Glands were incubated overnight at 4°C in primary antibody. They were then washed for 6 × 10 minutes in PBST before incubation with either Cy3- or Cy5-conjugated donkey anti-mouse secondary antibody (Jackson Laboratories) used at a dilution of 1 in 400 for 2 hours at room temperature. Glands were further washed in PBST for 6 × 10 min before mounting on slides using DAPI-containing Vectashield (Vector Laboratories). Imaging of glands was performed using a Zeiss Axioplan 2 scanning confocal microscope with an LSM510 laser module or a Zeiss 880 Airyscan system. Nuclear areas were measured using Axiovision freeware (Zeiss) as previously described [9].

### SC migration assay

The AGs of 16-day-old adult virgin males were dissected and fixed as described earlier. Glands were incubated in TRITC-phalloidin (Sigma-Aldrich; diluted 1:400 from a 1-mg/ml stock) for 1 hour and then washed three times for 5 minutes in PBST before mounting in DAPI-containing medium (Vectashield; Vector Laboratories). Glands were then examined by confocal microscopy as described earlier, and the number of proximally located SCs was counted.

### Analysis of SC secretion

CD63-GFP [14] was used instead of GFP-GPI [13] for the compartment pulse-labelling experiment because GFP-GPI fluorescence is poorly preserved after paraformaldehyde fixation, which is required for EdU detection. Males expressing CD63-GFP under temperature-inducible *dsx-GAL4* control [14] were multiply mated for 6 days at 25°C with 10 *w*^*1118*^ females on EdU-containing food. On day 7, the males were mated with five new *w*^*1118*^ females on EdU-containing food for 24 hours at 25°C. The males were isolated, and then a pulse of CD63-GFP expression was induced at 29°C for 24 hours on normal food. The AGs were then dissected and stained for EdU using the Click-iT Plus EdU kit. The number of CD63-GFP-labelled large compartments in each SC was counted in endoreplicated and nonendoreplicated cells.

### Detection of DNA replication using EdU

To detect DNA replication using the thymidine analogue EdU, either the Click-iT EdU imaging kit or the Click-iT Plus EdU imaging kit (Invitrogen) was used [74]. Adult flies were maintained on medium containing 0.2 mM EdU (ThermoFisher) from eclosion until dissection at 6 days. The EdU-containing medium was prepared by mixing standard cornmeal agar medium with a 10 mM stock solution (diluted in PBS per the manufacturer’s instruction). To detect EdU incorporation, dissected AGs were fixed in paraformaldehyde and processed as they were for immunohistochemistry. The Click-iT EdU reaction mix was prepared following the manufacturer’s instructions using a 1:400 dilution of a 2 mM stock of azide-fluor 555 (Sigma-Aldrich) dissolved in DMSO (this preparation was not required for the Click-iT Plus EdU kit). To label EdU-containing DNA in the sample, 200 μl of the reaction mix was added to the vials and left to incubate away from light for 30 minutes at 20°C. Glands were washed three times in 200 μl PBST and then resuspended in 200 μl PBS before mounting on coverslips using DAPI-containing mounting medium (Vectorshield; Vector Laboratories).

### Statistical analyses

We compared the mean SC:MC nuclear area across genotypes and controls. Having confirmed that the data were normally distributed by the Shapiro-Wilk test, we used one-way ANOVA and either Tukey’s or Sidak’s multiple-comparison posttest to identify significant changes. For nonparametric data, a Kruskal-Wallis test was performed with Dunn’s multiple-comparison test. Differences were deemed significant at a *p*-value < 0.05. Statistical analyses were performed using GraphPad Prism 7.0 or 8.0, (GraphPad Software, www.graphpad.com). Similar statistical analyses were performed to compare the mean proportion of SCs incorporating EdU across genotypes with control glands, whereas a two-tailed Mann-Whitney test was employed for the secretion data involving compartment counts.

## Supporting information

S1 Fig BMP signalling and the EcR synergise to regulate SC growth. Dissected AGs from 6-day-old males were stained with an antibody against Fasciclin3 to mark the apical outlines of SCs and neighbouring MCs (yellow) and with DAPI (blue nuclei). Selected SC nuclei are marked with red arrows and express GFP and other transgenes under esg^ts^F/O control. (A, B) RNAi-mediated knockdown of *Usp* has no effect on SC nuclear growth (B) compared with control (A). (D-G) Overexpression of the -A (D) and -B2 (F) isoforms of EcR has no effect on SC nuclear growth, but coexpression of these isoforms with Tkv^ACT^ synergistically promotes growth (E, G). (H) RNAi-mediated knockdown of a control gene, *ry*, had no effect on growth. (I, J) Histograms showing size of SC nuclei relative to MC nuclei in AGs in which SCs express different transgenes as above. Note that the EcR-B2-ΔC655.W650A construct was expressed in SCs using a temperature-inducible *dsx-GAL4* driver (see Materials and methods). Significance was assessed by one-way ANOVA with Tukey’s multiple-comparisons test. ***p < 0.001, *n* ≥ 9 (I), *n* ≥ 29 (J). Scale bars, 60 μm. Underlying data for this figure can be found in S1 Data. AG, accessory gland; BMP, bone morphogenetic protein; *dsx-GAL4*, *doublesex-GAL4*; EcR, ecdysone receptor; *esg*, *escargot*; esg^ts^F/O, the yeast transcription factor GAL4 expressed under the control of the promoter of the gene *esg* in a temperature-dependent fashion; GFP, green fluorescent protein; MC, main cell; RNAi, RNA interference; *ry*, *rosy*; SC, secondary cell; Tkv, Thick veins; *Usp*, Ultraspiracle.
(TIF)

S2 Fig BMP signalling, but not USP, regulates levels of the EcR protein in SCs. Images show the AG epithelium dissected from 6-day-old virgin males expressing nuclear GFP and other transgenes under esg^ts^F/O control and stained with a pan-EcR antibody (A-G) or anti-USP antibody (H,I). Nuclei are stained with DAPI (blue). Selected SC nuclei are marked with red arrows. (A, B) *Usp* knockdown (B) has no effect on EcR expression compared with control (A). (C-G) Overexpression of EcR-A (D) or EcR-B2 (F) does not appear to significantly alter EcR expression compared with controls (A). Coexpression of these isoforms with Tkv^ACT^ in SCs ([E] and [G], respectively) increases EcR expression in SCs compared with controls (A) and SCs expressing Tkv^ACT^ alone (C). (H,I) Immunostaining with an antibody that recognises USP reveals expression in the nuclei of control SCs (H), but absence of expression in the nuclei of SCs expressing an RNAi targeting *Usp* (I). Scale bars, 60 μm (A-G), 120 μm (H, I). AG, accessory gland; BMP, bone morphogenetic protein; EcR, ecdysone receptor; *esg*, *escargot*; esg^ts^F/O, the yeast transcription factor GAL4 expressed under the control of the promoter of the gene *esg* in a temperature-dependent fashion; GFP, green fluorescent protein; RNAi, RNA interference; SC, secondary cell; Tkv, Thick veins; USP, Ultraspiracle.
(TIF)

S3 Fig Repeated rejection of males by females does not affect SC nuclear size or 20-HE levels. (A) Histogram showing SC nuclear size in control virgin males and males rejected daily over a period of 6 days prior to isolation and analysis of accessory glands. (B) Histogram showing whole-animal titres of 20-HE in virgin and mated males and in males subjected to a female-rejection regime. Titres were significantly elevated in mated 6-day-old males compared with virgin controls but not after rejection. Significance was assessed by unpaired *t* test ([A]; *n* > 10) and by one-way ANOVA with Dunnett’s multiple-comparisons test (B). **p < 0.01, *n* ≥ 36 (A), *n* = 3 (B). Underlying data for this figure can be found in S1 Data. 20-HE, 20-hydroxyecdysone; SC, secondary cell.
(TIF)

S4 Fig BMP signalling does not regulate levels of the EcR protein in main cells. Images show the AG epithelium dissected from 6-day-old virgin males expressing nuclear GFP and other transgenes in main cells under Acp26Aa-GAL4 control and stained with a pan-EcR antibody. Note that GFP is also observed in the main cell cytoplasm when expressed at high levels in these cells. Nuclei are stained with DAPI (blue). Merge does not include DAPI channel for increased clarity. (A, B) Expression of Tkv^ACT^ in main cells (B), which do not normally express EcR (see control cells in [A]), does not affect EcR levels. (C-J) Expression of EcR-B1 (C), -B2 (E), -A (G), and -C (I) in main cells leads to accumulation of EcR in these cells, in contrast to SCs. Coexpression with Tkv^ACT^ does not appear to alter either the levels or subcellular localisation of EcR (D, F, H, J). Scale bars, 100 μm. Acp, AG protein; AG, accessory gland; BMP, bone morphogenetic protein; EcR, ecdysone receptor; GFP, green fluorescent protein; SC, secondary cell; Tkv, Thick veins.
(TIF)

S5 Fig BMP and EcR signalling function antagonistically to regulate SC migration. (A-G) Confocal images of whole accessory glands expressing nuclear GFP and other transgenes under esg^ts^F/O control. Panels show a single z-plane and therefore do not include all SCs in each gland; also, not all migrated SCs express GFP at sufficiently high levels to be detected at this magnification. In 16-day-old virgin males, an average of 11 ± 3 SCs expressing Tkv^ACT^ (B) and 4 ± 1 SCs expressing *EcR*-RNAi (C) migrate to the proximal end of the accessory gland (marked with white arrows), whereas no cells migrate from the distal tip (red arrows) in controls (A). Overexpression of EcR-B1 (D) or EcR-C (F) has no effect on SC migration. However, coexpression of EcR-B1 with Tkv^ACT^ (E) strongly suppresses cell migration, as does coexpression of Tkv^ACT^ with EcR-C (G). (H) Data analysed using the Kruskal-Wallis test with Dunn’s multiple-comparisons test. ****p < 0.0001, *n =* 15. Underlying data for this figure can be found in S1 Data. BMP, bone morphogenetic protein; EcR, ecdysone receptor; *esg*, *escargot*; esg^ts^F/O, the yeast transcription factor GAL4 expressed under the control of the promoter of the gene *esg* in a temperature-dependent fashion; GFP, green fluorescent protein; RNAi, RNA interference; SC, secondary cell; Tkv, Thick veins.
(TIF)

S1 Data Excel spreadsheet containing, in separate sheets, the underlying numerical data for panels in Figs 2, 3, 5 and 7–10 and S1, S3 and S5 Figs. (XLSX)

## Abbreviations

20-HE: 20-hydroxyecdysone
*Abd-B*: *Abdominal B*
Acp: AG protein
AF1: activation function 1
AG: accessory gland
AR: androgen receptor
BMP: bone morphogenetic protein
BrdU: bromodeoxyuridine
CHO: Chinese hamster ovary
CycE: Cyclin E
Dad: Daughters against Decapentaplegic
Dpp: Decapentaplegic
*ecd*: *ecdysoneless*
EcR: ecdysone receptor
EdU: 5-ethynyl-2′-deoxyuridine
*Eip74EF*: *Ecdysone-induced protein 74EF*
*esg*: *escargot*
esg^ts^F/O: the yeast transcription factor GAL4 expressed under the control of the promoter of the gene *esg* in a temperature-dependent fashion
Fas3: Fasciclin3
GFP: green fluorescent protein
MC: main cell
*Med*: *Medea*
NTD: N-terminal domain
P-Mad: phosphorylated Mothers against Decapentaplegic
PTTH: prothoracicotropic hormone
RNAi: RNA interference
RT-PCR: reverse-transcription PCR
*ry*: *rosy*
SC: secondary cell
*shd*: *shade*
*spo*: *spook*
TGF-β: transforming growth factor β
Tkv: Thick veins
tub-GAL80^ts^: a temperature-sensitive form of the GAL4 inhibitor GAL80 expressed under the control of the *tubulin* gene promoter
USP: Ultraspiracle
Wit: Wishful thinking
