# Supplementary figures and images for: Mating induces switch from hormone-dependent to hormone-independent steroid receptor–mediated growth in Drosophila secondary cells
Source: PLoS Biol. 2019 Oct 7;17(10):e3000145. doi: 10.1371/journal.pbio.3000145 (PMC6797231; doi:10.1371/journal.pbio.3000145)

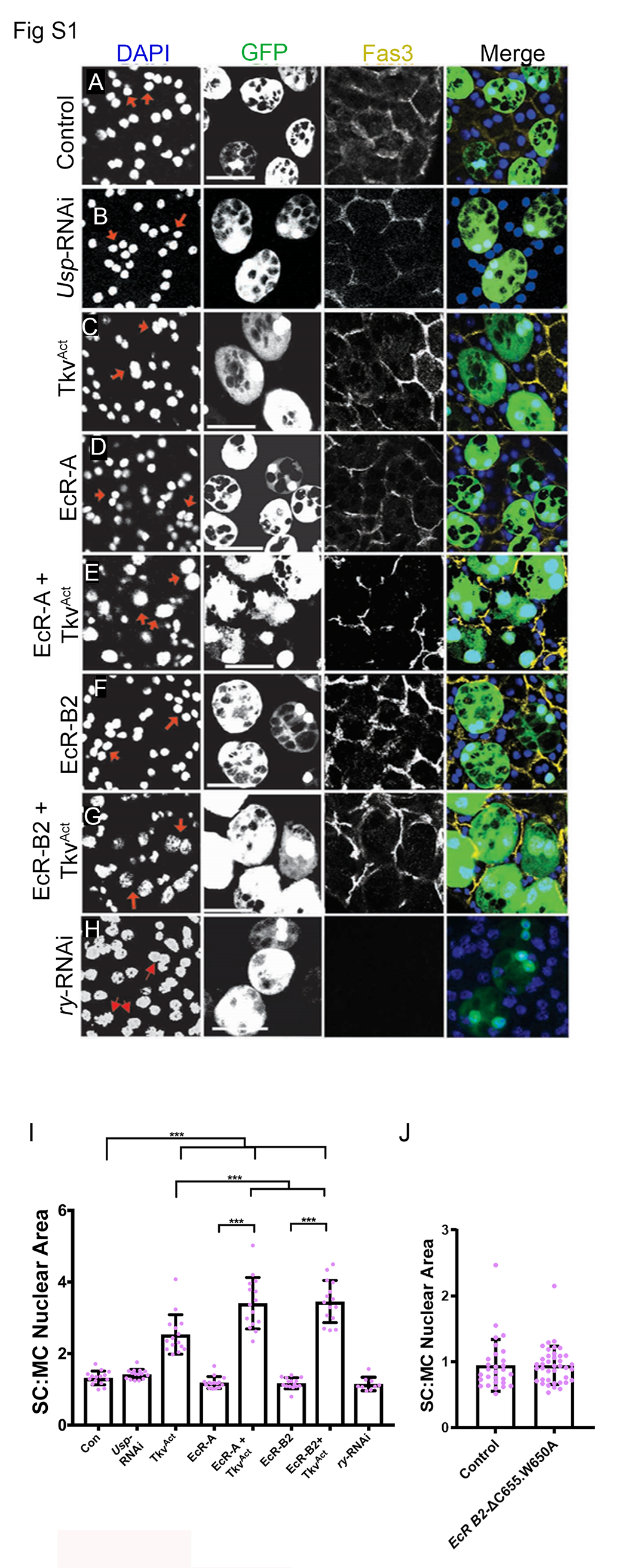

Supplement: S1 Fig — Dissected AGs from 6-day-old males were stained with an antibody against Fasciclin3 to mark the apical outlines of SCs and neighbouring MCs (yellow) and with DAPI (blue nuclei). Selected SC nuclei are marked with red arrows and express GFP and other transgenes under esgtsF/O control. (A, B) RNAi-mediated knockdown of Usp has no effect on SC nuclear growth (B) compared with control (A). (D-G) Overexpression of the -A (D) and -B2 (F) isoforms of EcR has no effect on SC nuclear growth, but coexpression of these isoforms with TkvACT synergistically promotes growth (E, G). (H) RNAi-mediated knockdown of a control gene, ry, had no effect on growth. (I, J) Histograms showing size of SC nuclei relative to MC nuclei in AGs in which SCs express different transgenes as above. Note that the EcR-B2-ΔC655.W650A construct was expressed in SCs using a temperature-inducible dsx-GAL4 driver (see Materials and methods). Significance was assessed by one-way ANOVA with Tukey’s multiple-comparisons test. ***p < 0.001, n ≥ 9 (I), n ≥ 29 (J). Scale bars, 60 μm. Underlying data for this figure can be found in S1 Data. AG, accessory gland; BMP, bone morphogenetic protein; dsx-GAL4, doublesex-GAL4; EcR, ecdysone receptor; esg, escargot; esgtsF/O, the yeast transcription factor GAL4 expressed under the control of the promoter of the gene esg in a temperature-dependent fashion; GFP, green fluorescent protein; MC, main cell; RNAi, RNA interference; ry, rosy; SC, secondary cell; Tkv, Thick veins; Usp, Ultraspiracle. (TIF) [file pbio.3000145.s001.tif]

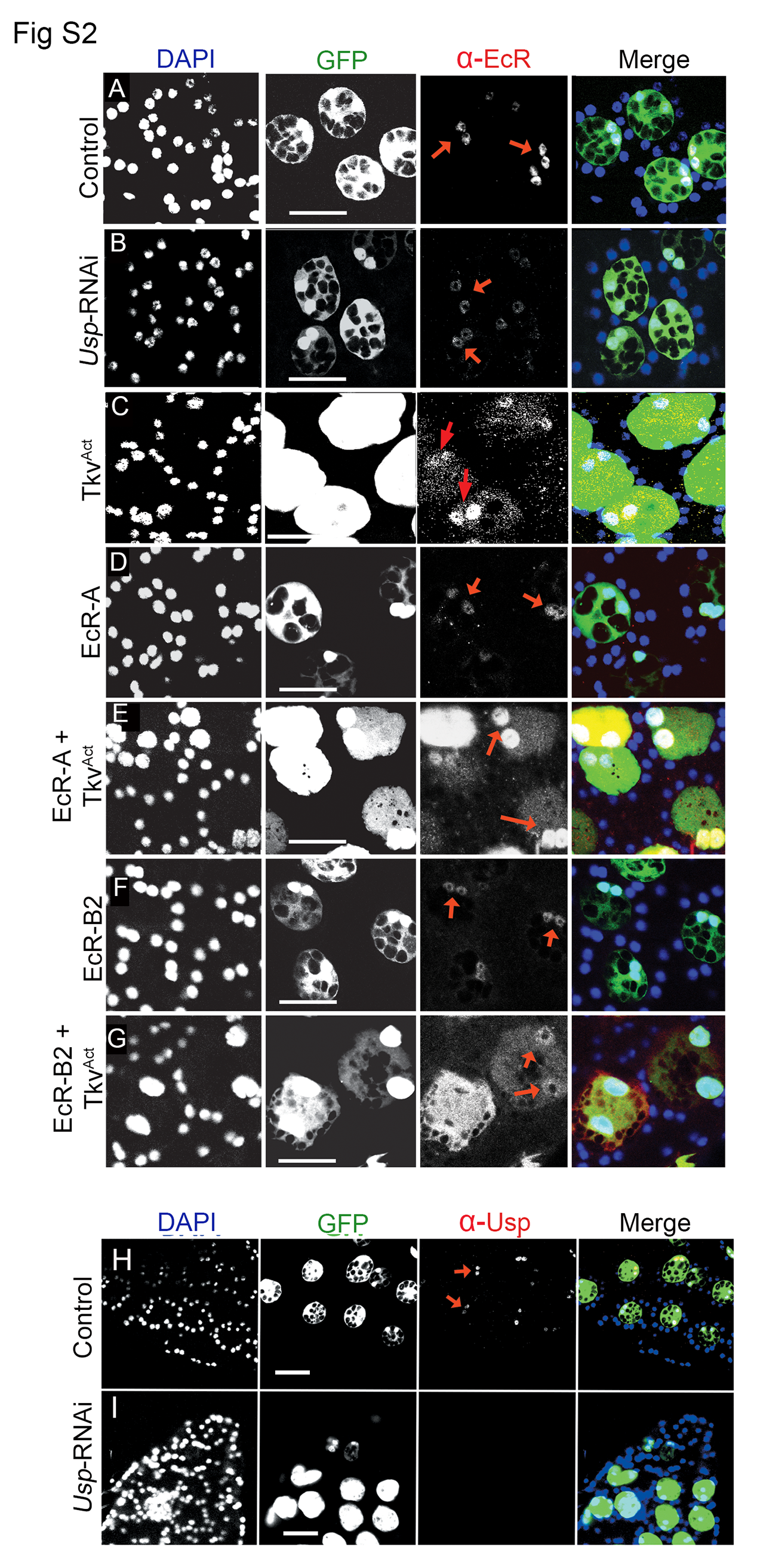

Supplement: S2 Fig — Images show the AG epithelium dissected from 6-day-old virgin males expressing nuclear GFP and other transgenes under esgtsF/O control and stained with a pan-EcR antibody (A-G) or anti-USP antibody (H,I). Nuclei are stained with DAPI (blue). Selected SC nuclei are marked with red arrows. (A, B) Usp knockdown (B) has no effect on EcR expression compared with control (A). (C-G) Overexpression of EcR-A (D) or EcR-B2 (F) does not appear to significantly alter EcR expression compared with controls (A). Coexpression of these isoforms with TkvACT in SCs ([E] and [G], respectively) increases EcR expression in SCs compared with controls (A) and SCs expressing TkvACT alone (C). (H,I) Immunostaining with an antibody that recognises USP reveals expression in the nuclei of control SCs (H), but absence of expression in the nuclei of SCs expressing an RNAi targeting Usp (I). Scale bars, 60 μm (A-G), 120 μm (H, I). AG, accessory gland; BMP, bone morphogenetic protein; EcR, ecdysone receptor; esg, escargot; esgtsF/O, the yeast transcription factor GAL4 expressed under the control of the promoter of the gene esg in a temperature-dependent fashion; GFP, green fluorescent protein; RNAi, RNA interference; SC, secondary cell; Tkv, Thick veins; USP, Ultraspiracle. (TIF) [file pbio.3000145.s002.tif]

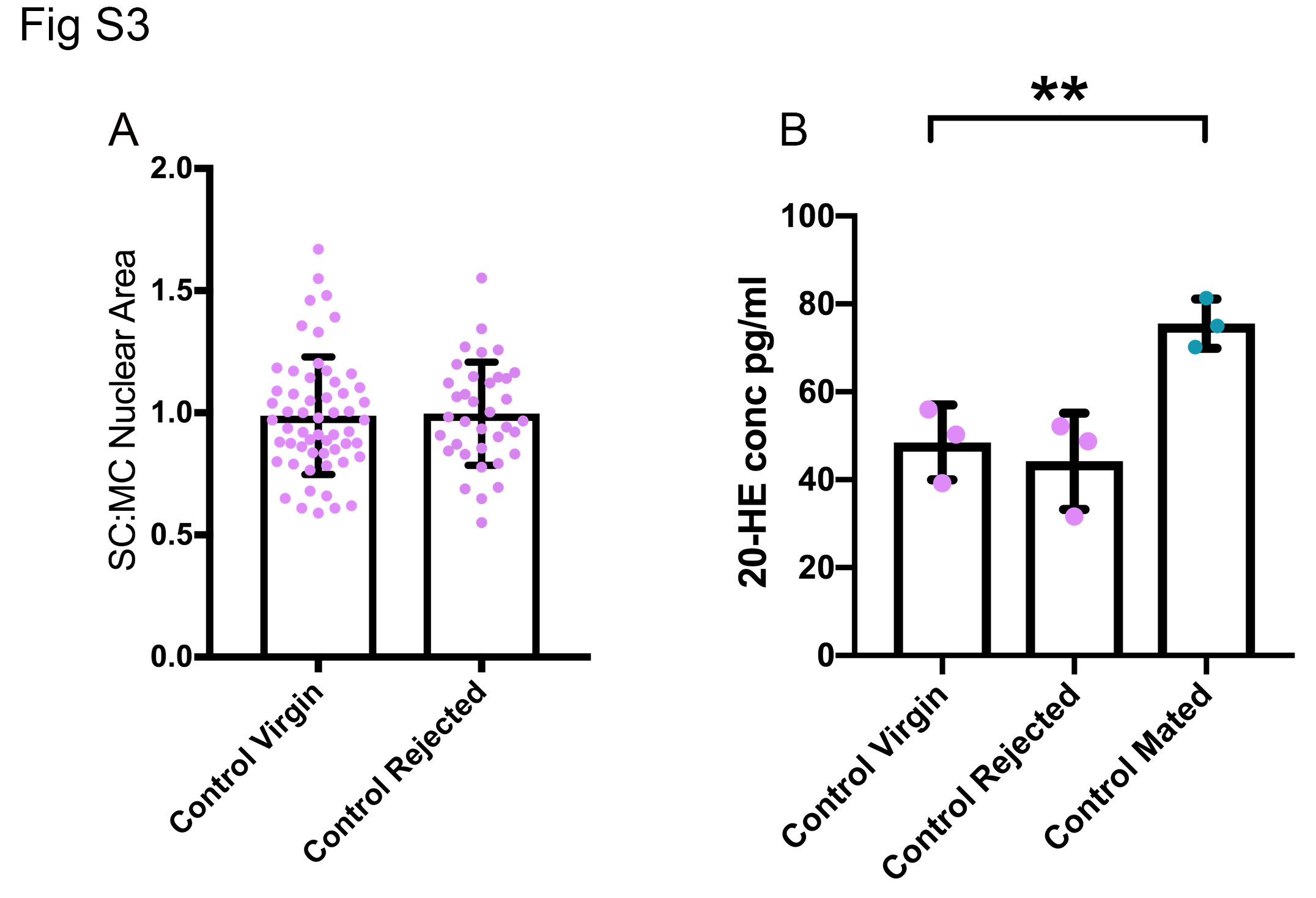

Supplement: S3 Fig — (A) Histogram showing SC nuclear size in control virgin males and males rejected daily over a period of 6 days prior to isolation and analysis of accessory glands. (B) Histogram showing whole-animal titres of 20-HE in virgin and mated males and in males subjected to a female-rejection regime. Titres were significantly elevated in mated 6-day-old males compared with virgin controls but not after rejection. Significance was assessed by unpaired t test ([A]; n > 10) and by one-way ANOVA with Dunnett’s multiple-comparisons test (B). **p < 0.01, n ≥ 36 (A), n = 3 (B). Underlying data for this figure can be found in S1 Data. 20-HE, 20-hydroxyecdysone; SC, secondary cell. (TIF) [file pbio.3000145.s003.tif]

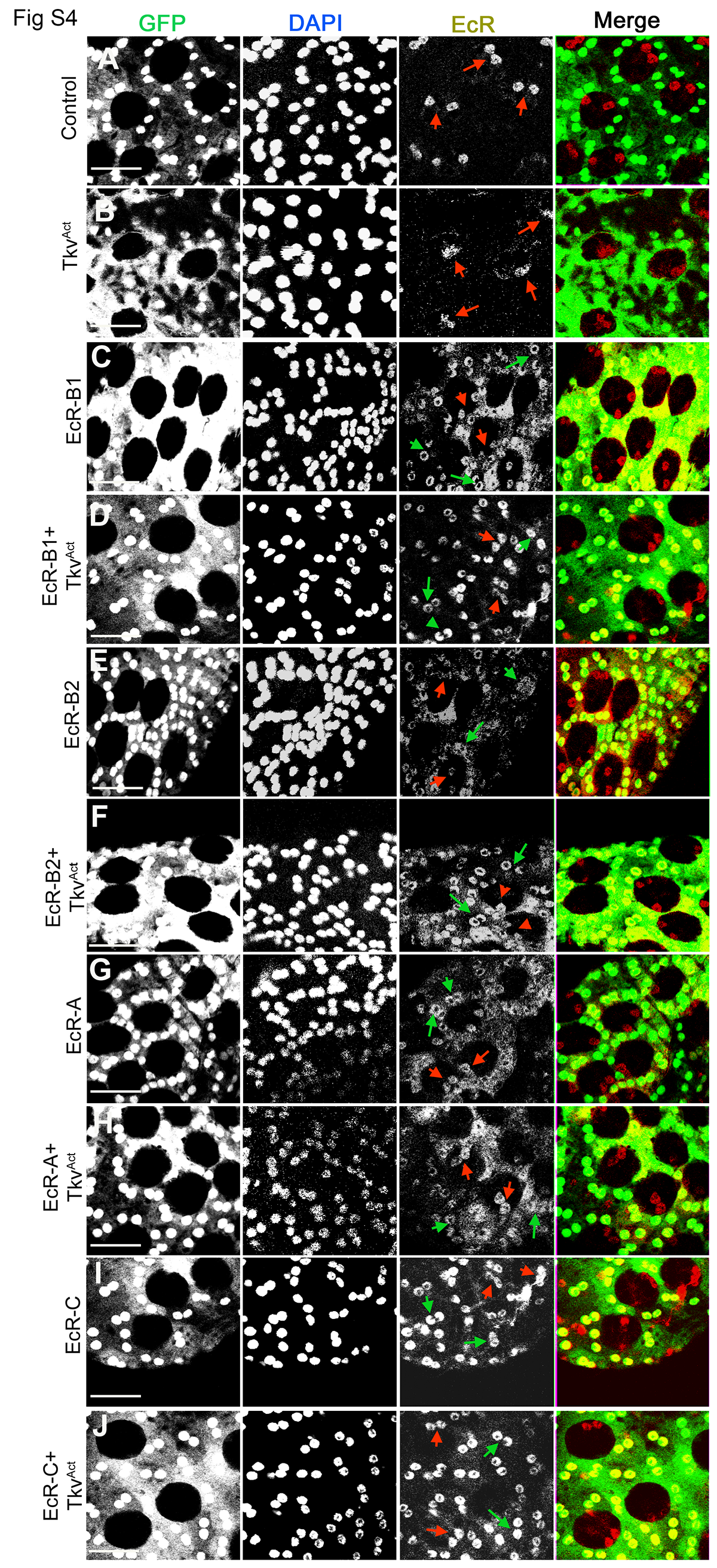

Supplement: S4 Fig — Images show the AG epithelium dissected from 6-day-old virgin males expressing nuclear GFP and other transgenes in main cells under Acp26Aa-GAL4 control and stained with a pan-EcR antibody. Note that GFP is also observed in the main cell cytoplasm when expressed at high levels in these cells. Nuclei are stained with DAPI (blue). Merge does not include DAPI channel for increased clarity. (A, B) Expression of TkvACT in main cells (B), which do not normally express EcR (see control cells in [A]), does not affect EcR levels. (C-J) Expression of EcR-B1 (C), -B2 (E), -A (G), and -C (I) in main cells leads to accumulation of EcR in these cells, in contrast to SCs. Coexpression with TkvACT does not appear to alter either the levels or subcellular localisation of EcR (D, F, H, J). Scale bars, 100 μm. Acp, AG protein; AG, accessory gland; BMP, bone morphogenetic protein; EcR, ecdysone receptor; GFP, green fluorescent protein; SC, secondary cell; Tkv, Thick veins. (TIF) [file pbio.3000145.s004.tif]

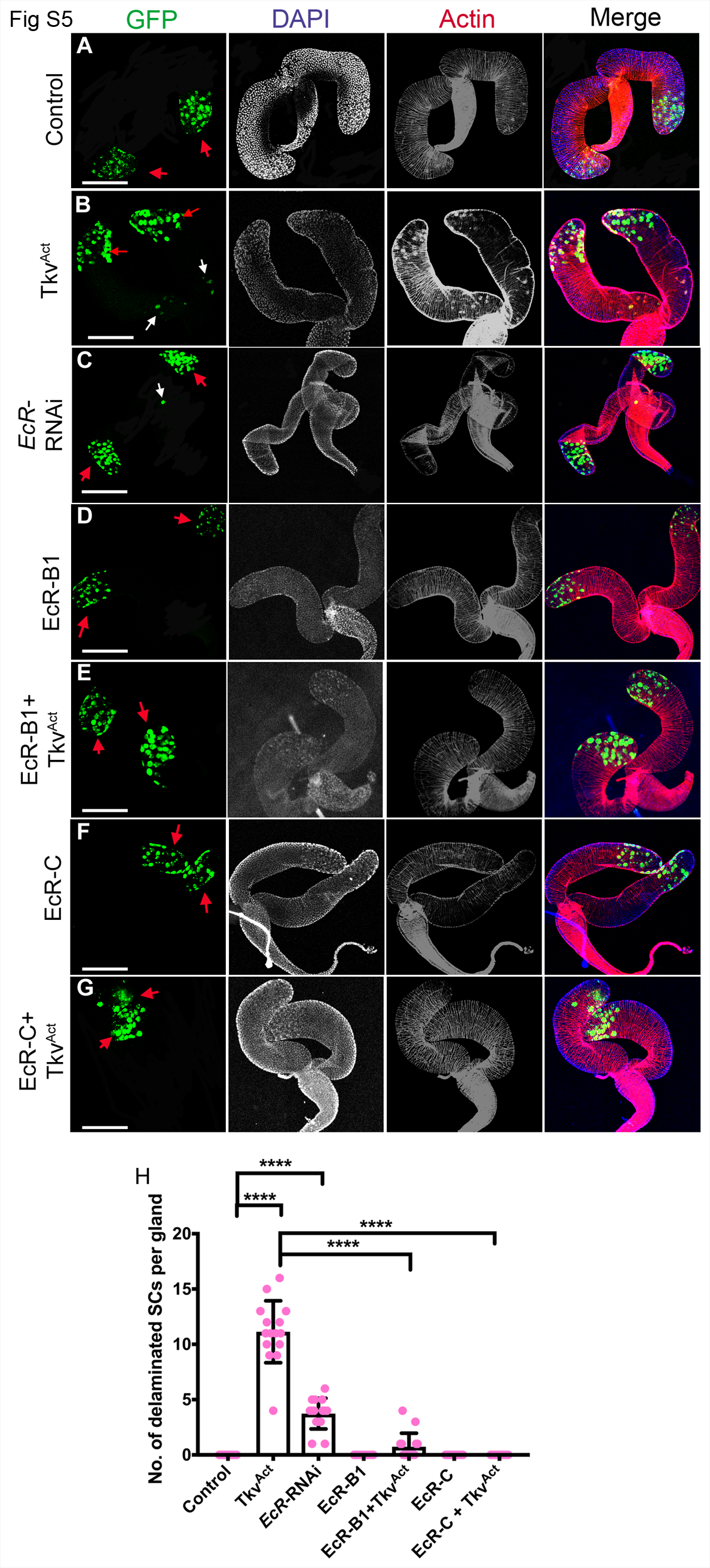

Supplement: S5 Fig — (A-G) Confocal images of whole accessory glands expressing nuclear GFP and other transgenes under esgtsF/O control. Panels show a single z-plane and therefore do not include all SCs in each gland; also, not all migrated SCs express GFP at sufficiently high levels to be detected at this magnification. In 16-day-old virgin males, an average of 11 ± 3 SCs expressing TkvACT (B) and 4 ± 1 SCs expressing EcR-RNAi (C) migrate to the proximal end of the accessory gland (marked with white arrows), whereas no cells migrate from the distal tip (red arrows) in controls (A). Overexpression of EcR-B1 (D) or EcR-C (F) has no effect on SC migration. However, coexpression of EcR-B1 with TkvACT (E) strongly suppresses cell migration, as does coexpression of TkvACT with EcR-C (G). (H) Data analysed using the Kruskal-Wallis test with Dunn’s multiple-comparisons test. ****p < 0.0001, n = 15. Underlying data for this figure can be found in S1 Data. BMP, bone morphogenetic protein; EcR, ecdysone receptor; esg, escargot; esgtsF/O, the yeast transcription factor GAL4 expressed under the control of the promoter of the gene esg in a temperature-dependent fashion; GFP, green fluorescent protein; RNAi, RNA interference; SC, secondary cell; Tkv, Thick veins. (TIF) [file pbio.3000145.s005.tif]
